# Crosstalk in the darkness: bulb vernalization activates meristem transition via circadian rhythm and photoperiodic pathway

**DOI:** 10.1186/s12870-020-2269-x

**Published:** 2020-02-17

**Authors:** Tomer E. Ben Michael, Adi Faigenboim, Einat Shemesh-Mayer, Itzhak Forer, Chen Gershberg, Hadass Shafran, Haim D. Rabinowitch, Rina Kamenetsky-Goldstein

**Affiliations:** 1 Institute of Plant Sciences, ARO, The Volcani Center, Rishon LeZion, Israel; 2 Robert H. Smith Faculty of Agricultural, Food, and Environmental Quality Sciences, The Hebrew University of Jerusalem, Rehovot, Israel

**Keywords:** *Allium sativum*, Bulbing, Flowering, Reproductive meristem, Low temperature

## Abstract

**Background:**

Geophytes possess specialized storage organs - bulbs, tubers, corms or rhizomes, which allow their survival during unfovarable periods and provide energy support for sprouting and sexual and vegetative reproduction. Bulbing and flowering of the geophyte depend on the combined effects of the internal and external factors, especially temperature and photoperiod. Many geophytes are extensively used in agriculture, but mechanisms of regulation of their flowering and bulbing are still unclear.

**Results:**

Comparative morpho-physiological and transcriptome analyses and quantitative validation of gene expression shed light on the molecular regulation of the responses to vernalization in garlic, a typical bulbous plant. Long dark cold exposure of bulbs is a major cue for flowering and bulbing, and its interactions with the genetic makeup of the individual plant dictate the phenotypic expression during growth stage. Photoperiod signal is not involved in the initial nuclear and metabolic processes, but might play role in the later stages of development, flower stem elongation and bulbing. Vernalization for 12 weeks at 4 °C and planting in November resulted in flower initiation under short photoperiod in December–January, and early blooming and bulbing. In contrast, non-vernalized plants did not undergo meristem transition. Comparisons between vernalized and non-vernalized bulbs revealed ~ 14,000 differentially expressed genes.

**Conclusions:**

Low temperatures stimulate a large cascades of molecular mechanisms in garlic, and a variety of flowering pathways operate together for the benefit of meristem transition, annual life cycle and viable reproduction results.The circadian clock appears to play a central role in the transition of the meristem from vegetative to reproductive stage in bulbous plant, serving as integrator of the low-temperature signals and the expression of the genes associated with vernalization, photoperiod and meristem transition. The reserved photoperiodic pathway is integrated at an upstream point, possibly by the same receptors. Therefore, in bulb, low temperatures stimulate cascades of developmental mechanisms, and several genetic flowering pathways intermix to achieve successful sexual and vegetative reproduction.

## Background

Perennial plant species carefully time their flowering with seasonal changes to ensure and maximize reproductive success. Flowering is induced, initiated and promoted both by internal and environmental cues, such as physiological age, gibberellin synthesis, photoperiod, and/or temperatures. The combined effect of two or more of these agents results in an integrated regulatory network that controls flowering time and quality [4, 89].

Despite the considerable genetic differences between and within species [9, 10, 18, 29], there is a great physiological similarity among plants from the temperate zone as many of them depend on vernalization for floral induction. Accumulation of chilling hours regulates essential changes in water status, in hormonal balance, in respiration and in carbohydrate mobilization [39], with a consequent increase in plant receptivity to changes in day length in the following spring [4].

Dependence on low temperatures evolved several times in the history of plants, with the consequent involvement of a number of regulatory mechanisms [4, 10, 40, 74]. For instance, *Arabidopsis* remains vegetative when *FRIGIDA (FRI)* upregulates the floral repressor *FLOWERING LOCUS C (FLC),* which in turn downregulates the floral integrators *FT, FD*, and *SOC1*. Cold induction downregulates *FLC,* thus enabling the expression of floral integrators and consequently the meristem transition from the vegetative to the reproductive state [44, 60, 79, 80]. An alternative regulating mechanism is common in the monocot cereals, where *FT*-homolog *VERNALIZATION 3 (VRN3)* is repressed by *VRN2*. Following cold induction, *VRN1* represses *VRN2* expression in the leaves, thus enabling the expression of *VRN3* and the consequent transition of the meristem [74]. In bulb onion, *FT*-like genes control the initiation of both bulbing and flowering [45]. Under short photoperiod, high *AcFT4* expression inhibits bulb formation by repressing *AcFT1.* This genotype-specific inhibitory effect gradually weakens when days elongate, and thereafter the inductive photoperiod downregulates *AcFT4*, with the consequent expression of bulb-promoting *AcFT1*. On the other hand, low temperatures in storage and/or in the field promote the up-regulation of *AcFT2*, which encodes for flowering in the spring/summer. Vernalization effects on the upregulation of *FT*-like sequences (*LiFTL*) were also reported for *Lilium longiflorum* [47, 49]. In tulip, *TgFT2* is considered to act as florigen, whereas *TgFT1* and *TgFT3* may have bulb-specific functions [47].

Unlike vernalization, the molecular mechanism of the photoperiodic pathway evolved in early times and is well conserved in plants [4, 91]. It involves stabilizing/destabilizing balance between photoreceptors, with the consequent timely induction and initiation of flowering by the expression of *CONSTANS* (*CO*), which is regulated by both*, GIGANTEA (GI)* and the circadian rhythm [84, 91].

In many plant species, flowering induction and initiation require sequential and combined effects of vernalization and photoperiod. Hence a ‘memory’ of the vernalization effect is maintained by epigenetic mechanisms [8, 86]. In *Arabidopsis,* these chromatin modifications suppress the floral repressor *FLC* [16, 86], while in cereals they upregulate the floral activator *VRN1* [67, 90]. It is generally accepted that for both mono- and dicots, *FT*s homologs act as the main genetic floral integrator of various pathways [74].

Geophytes are defined as plants with underground storage organs - bulbs, tubers, corms or rhizomes. In climates with environmental extremes, storage organs assist in survival during unfovarable periods and for providing energy support for sprouting and sexual and vegetative reproduction. Their life cycle consists of complex environment-dependent annual sequences, where induction and initiation of flowering and bulbing are tightly controlled by the surrounding conditions [34]. Many geophyte species are extensively used in agriculture, and deep knowledge of their physiology is readily available, while main mechanisms of molecular regulation is still unclear [35].

The annual development cycle of typical bulbous geophyte garlic (*Allium sativum* L.), is well adapted to the environmental changes common in its center of origin in Central Asia [36]. Hence, both bulbing and flowering are regulated by the combined effects of temperature and photoperiod [38, 58, 71, 72, 82, 94]. In bolting genotypes, low temperatures induce the transition of the meristem from vegetative to the reproductive state, with the consequent differentiation of inflorescence, scape elongation, and growth of axillary buds. In the early summer, when temperatures increase and days get longer, blooming occurs and mature compound bulbs enter dormancy.

Pre-planting cold treatment and/or winter temperatures significantly affect the developmental regulation and phenotypic expression of growing garlic plants, their bulbing and bulb quality, as well as its reproductive development [7]. Extended vernalization strongly expedite vegetative growth, early meristem transition and bulbing, thus resulting in the development of only a few axillary buds (cloves) and consequently in small bulbs and inflorescences [5, 7, 26, 76, 95].

It is commonly accepted that photoperiod is the dominant environmental factor that controls stem elongation and bulbing in both onion and garlic [38, 70]. However, our recent findings show that garlic pre-planting storage at 4 °C for 12 weeks resulted in photoperiod-independent meristem transition already in mid-winter, in December–January, when day length is shortest [7]. The present study aims to identify the molecular mechanisms involved in the regulation of responses to low temperature in a flowering garlic genotype. Furthermore, by comparing gene expression in plants exposed to long and short vernalization with non-vernalized plants, we obtained molecular evidence for flowering induction during the bulb cold storage.

## Results

### Morpho-physiological analysis

**Fig. 1:**
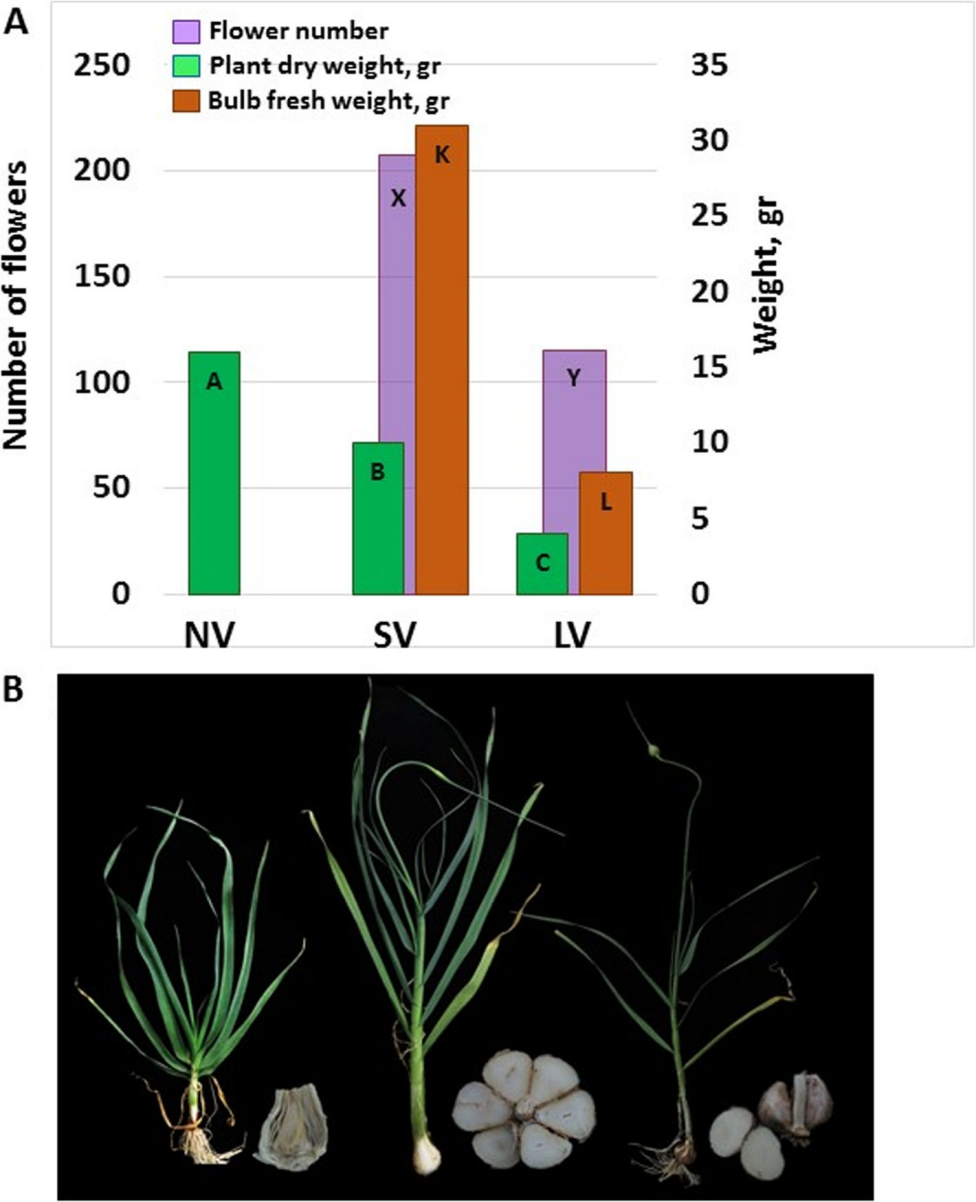
Effect of pre-planting vernalization on the development of #87 garlic plants. Planting and harvest dates were November 11th, 2015 and June–July 2016, respectively. **a** Plant and bulb weight and the number of flowers/inflorescence at the end of the growing season. **b** Images of intact plants in mid-season 140 DAP, March 30th and newly produced mature bulbs after harvest

Plants produced from the non-vernalized (NV) bulbs maintained a vegetative habitus and continuously produced new leaves throughout the growing period, up to 20 leaves in June [7]. In these plants, the apical meristem did not shift to floral initiation, apical dominance did not weaken and lateral meristems did not differentiate into leaves or cloves. Dry weight of aboveground parts reached 17 g, as compared with 11–12 and 3-4 g for the plants raised after short (SV) and long vernalization (LV), respectively (Fig. 1a).

In comparison, plants raised from vernalized bulbs lost their apical dominance a number of weeks after planting, developed reproductive organs and cloves and yielded dormant bulbs (Fig. 1b). In plants raised after SV, the short vernalization did not suffice for induction of meristem transition prior to planting, but at the end of March, 140 days after planting (DAP), apical dominance weakened and reproductive meristem was observed. These plants produced 17–19 leaves prior to flowering in May, 185 DAP, and developed approximately 160 flowers per inflorescence. On harvest, 230 DAP, bulbs had 6–7 cloves, and mean bulb weight was 30 g (Fig. 1).

In LV plants, meristem transition was visible already early in January ~ 50 DAP, after the development of 7–8 leaves (Fig. 1). The inflorescences produced 100–120 flowers, and the bulbs had only 2–3 cloves. Bulb maturation occurred on 200 DAP, and mean bulb weight was 8 g (Fig. 1). Therefore, the cold units accumulated during the long vernalization sufficed for initiation of both flowering and bulbing in clone #87.

### Global transcriptome analysis

RNA samples taken from clove apical meristems were used for the construction of pre-planting transcriptomes. The high-throughput parallel RNA-Seq libraries served for building cDNA libraries with 36–42 million 100-bp pair-end reads. Quality trimming and filtration resulted in 33–39 million clean reads. Together with the fertile garlic data (NCBI bioproject PRJNA243415; [37]) these reads were assembled using Trinity software, thus generated 112,388 trinity ‘genes’ with an average length of 1.178 bp and N50 of 1812 bp. About 90% of the clean reads were mapped to the *A. sativum* transcriptome catalog.

**Fig. 2:**
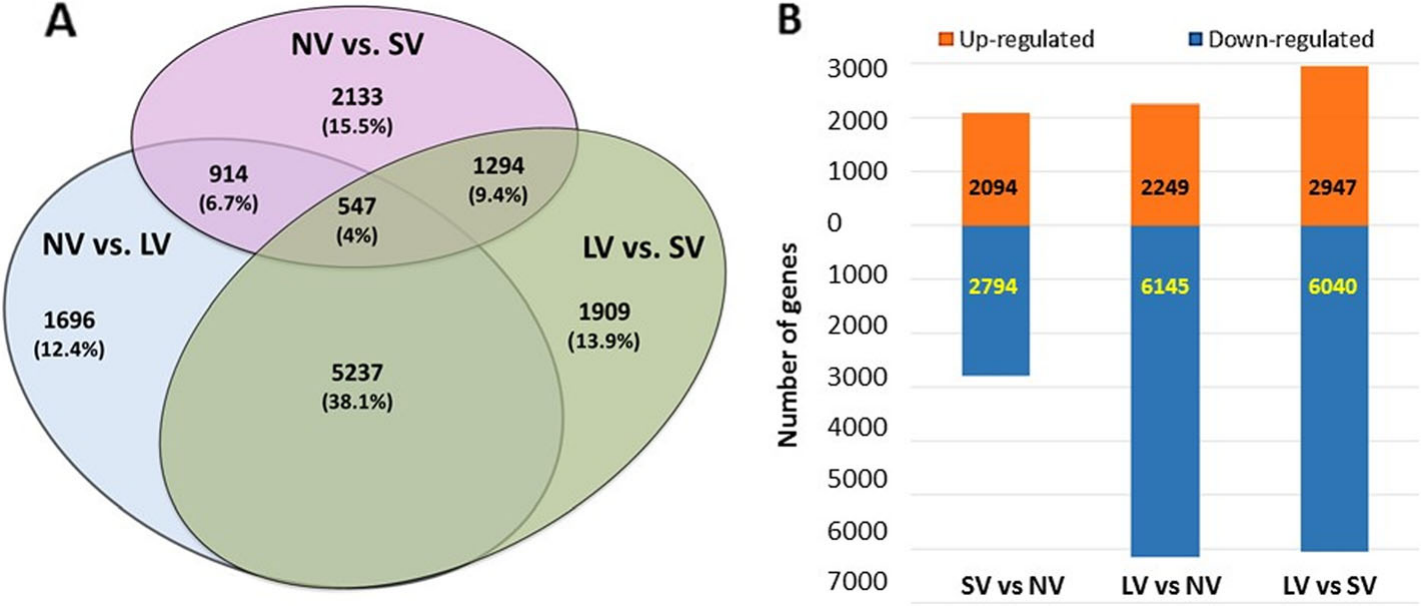
Effect of pre-planting vernalization treatments on mRNA expression in garlic meristems. Numbers indicate the number of differentially expressed genes (DEGs) and their share among the ~ 14,000 DEGs in all comparisons. **a** Venn diagram. Numbers represent common and specific DEGs in each comparison between treatments; **b** Number of up- and downregulated genes in three pairwise comparisons at the end of storage treatments

Comparisons between pairs of treatments revealed that only 4% of the detected ~ 14,000 differentially expressed genes (DEGs) were present in all comparisons. Comparisons LV/NV and LV/SV shared the highest number of common DEGs (38%) (Fig. 2a). Long cold exposure resulted in the highest number of differences in transcription activity, in both up- and down-regulated genes. Moreover, in all comparisons, the number of down-regulated genes was considerably higher than that of up-regulated ones (Fig. 2b).

**Fig. 3:**
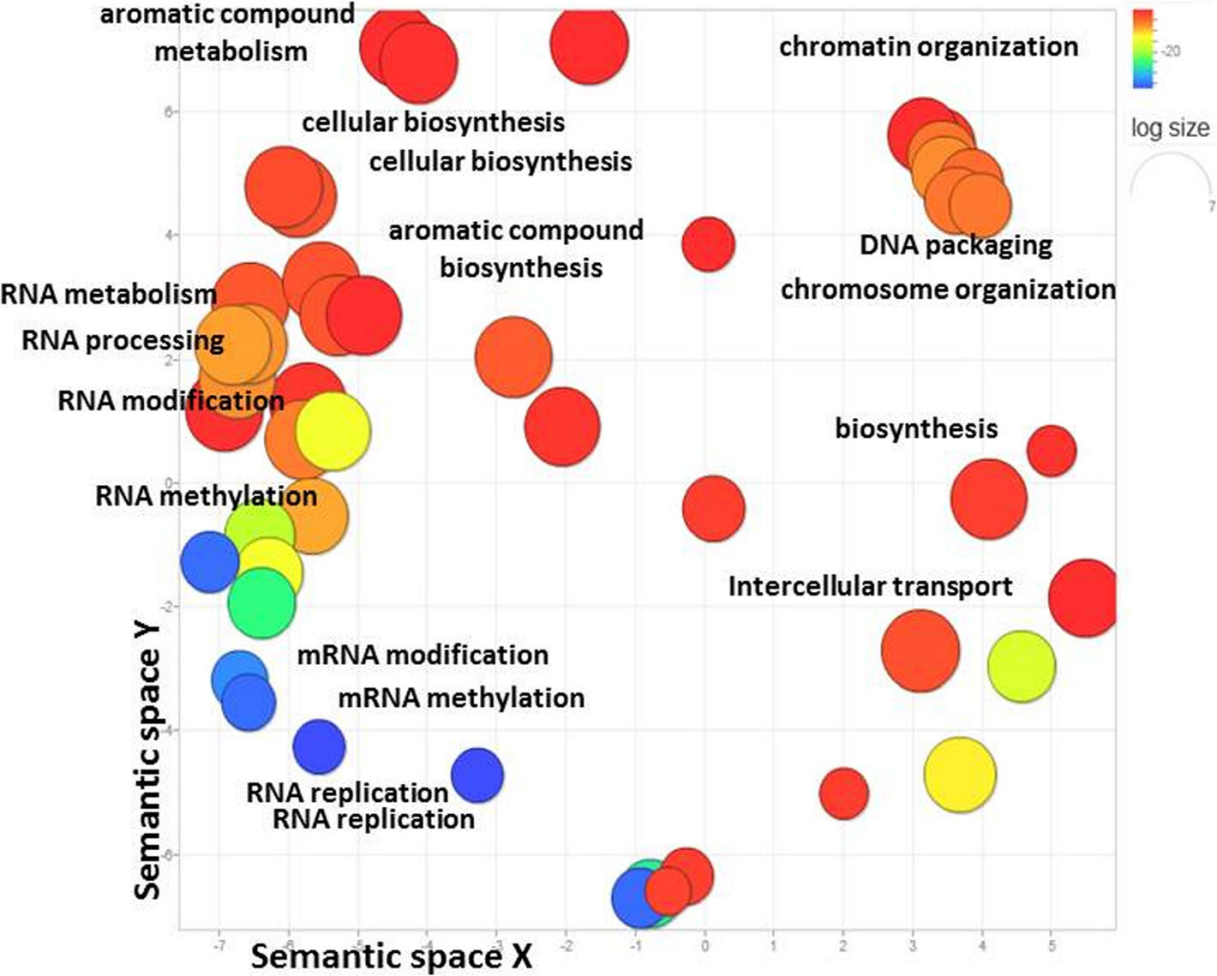
GO term distribution of 547 DEGs shared by all comparisons between vernalization treatments, as revealed by Blast2GO and REVIGO algorithms. Represented by circles, GO terms were plotted in agreement with semantic similarities to other GO terms (adjoining circles are most closely related). Main patterns are related to the chromatin organization, RNA replication, regulation of gene expression and methylation. Circle sizes are proportional to the frequency of the GO terms within each cluster, and circles’ color indicates the log10 corrected *P*-value for enrichment

Functional GO analysis of the 547 DEGs (4% of the total number) shared by all comparisons (Fig. 2a) revealed three main domains of activity - RNA methylation, chromatin organization, and biosynthesis of aromatic compounds (Fig. 3). These metabolic processes play general developmental roles towards the end of garlic dormancy, sprouting and meristem identity affected by vernalization.

**Fig. 4:**
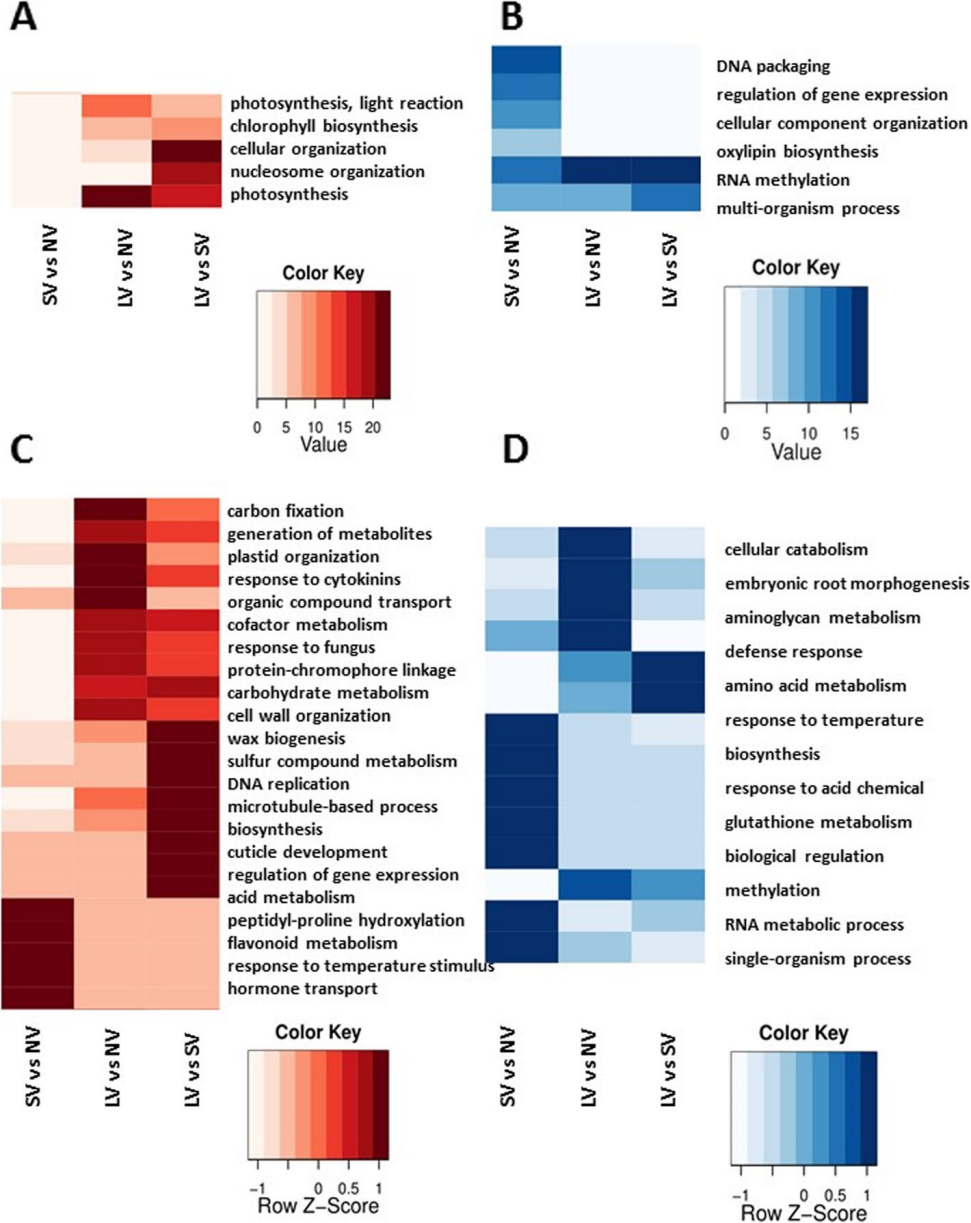
Overview of the GO enrichment analysis obtained from the transcriptome data of the effect of vernalization in garlic. Output GO enrichment analysis was performed by comparing data from NV – no vernalization, SV – short vernalization, LV – long vernalization of #87 garlic. The GO terms listed on the left-hand side, in red, are specifically overrepresented in the up-regulated genes. On the right-hand side, in blue, GO terms specifically overrepresented in the down-regulated genes. **a** and **b** represent corrected *P*-value < 4.8e^− 8^, **c** and **d** corrected *P*-value 7.5e^− 6^ to 0.05 (at least in one comparison). Corrected *P*-values were log10 transformed

Using GO algorithm, we analyzed the biological process functions of up- and down-regulated genes, in all comparisons (Fig. 4). For SV/NV comparison, the GO enrichment showed over-representation of the domains associated with hormone transport, response to temperature stimulus, and flavonoid metabolism (Fig. 4a, c), whereas chromatin organization, DNA packaging, regulation of gene expression, metabolic processes and biosynthesis, were down-regulated (Fig. 4b, d). The LV/NV comparison revealed strong over-represented domains associated with photosynthesis, carbon fixation, response to biotic stimulus and cytokinin, plastid organization, and light reaction (Fig. 4a, c). In contrast, function domains involved in cellular catabolism, defense response, and RNA metabolism were clearly down-regulated in LV cloves (Fig. 4b, d). Finally, the LV/SV comparison analysis showed upregulation of 2947 genes (Fig. 2a), associated with biosynthesis, chromatin organization, DNA modification and methylation, carbohydrate metabolism, photosynthesis and response to light, and biological processes related to photoperiodism, photosynthesis, response to external stimuli and auxin influx were abundant (Fig. 4a, c). The cellular component organization was extremely over-represented, whereas genes coding for a response to temperature stimulus and to RNA methylation were down-regulated (Fig. 4b).

### Data mining for functional analysis

Our results indicate that differential growth performance of plants is associated with expression of genes involved in meristem transition. To verify this assumption, we performed data-mining in the DEGs list, searching for gene annotations in the databases using the keywords: flowering, meristem transition, photoperiod, circadian clock and vernalization. Further, we analysed the literature data on specific gene families or genes with higher complexity, e.g., *FT* family, in order to compare garlic with model and non-model plant species and assess the meristem identitiy pathways.

Consequently, we compiled a list of 74 DEGs associated with determination of meristem identity (Additional file 5: Table S2).

### Gene co-expression networks (GCNs)

**Fig. 5:**
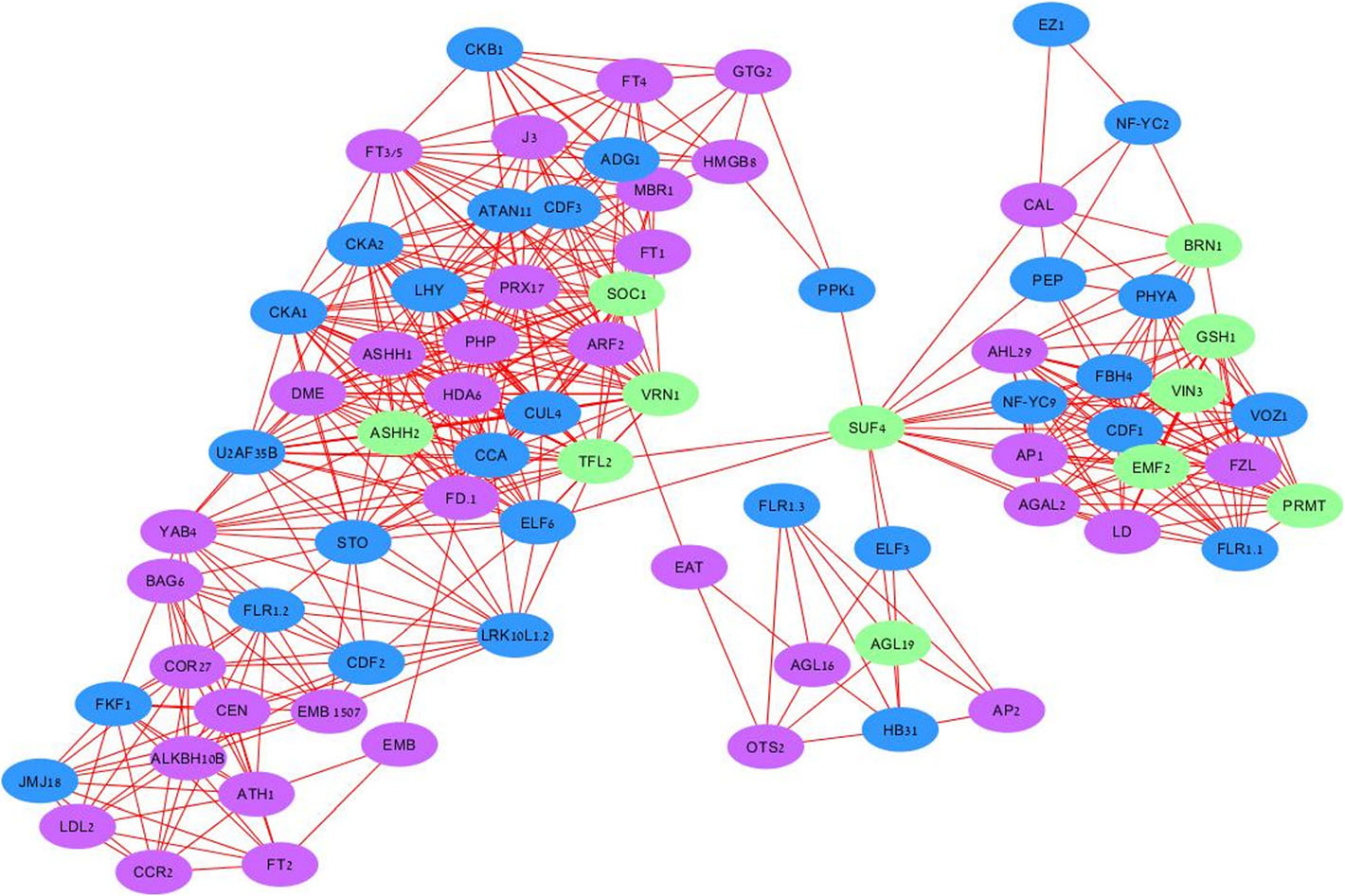
Positive co-expression of garlic genes associated with vernalization (green), photoperiod (blue) pathway and meristem transition (purple). Data from three vernalization treatments were analyzed using the network-drawing software Cytoscape [81]. Pearson correlation value higher than 0.9

Gene co-expression networks were constructed as the integration of multiple expression datasets by connecting genes with similar patterns of expression across treatments to reveal the specific biological context of positive and negative gene linkages in the apical meristem. In positive GCN representation, at least three main modules were identified, and co-expression was found between genes involved in vernalization and photoperiod pathways, and in those annotated as genes involved in meristem transition (Fig. 5). Vernalization-associated genes (marked green) are present in all three modules and are closely linked with genes annotated to photoperiod response (marked blue) and to meristem identity genes (marked purple). In general, genes associated with a light reception, photoperiod response and circadian clock (Additional file 5: Table S2) represent a major part of the positive GCN and allied with the meristem transition network. A garlic homolog of *SUPPRESSOR OF FRIGIDA 4 (SUF4)*, associated in *Arabidopsis* with activation of *FLC* expression, was found to be centrally positioned in the positive GCN. Hence, it seems to play a conserved role in integrating flowering-related pathways (Fig. 5).

Our analyses also revealed three *FLOR1* (*FLR1*) homologs, associated with long photoperiod pathway of meristem transition. *FLR1* homologs with slightly different sequences were present in each of the major modules, suggesting their possible involvement in flowering through alternative splicing. *SALT TOLERANCE (STO)*, strongly associated with photoperiod response and flowering transition, is another gene of interest. It is located in the center of the largest GCN module, connecting two sub-modules.

The *FT* family of genes (*FT1, FT2, FT4,* and *FT3/5*) is centrally located in this network, but with no direct links connect between them. *FT2* co-expresses with meristem-transition genes *CCR2, EMB, COR27*, while *FT1* and *FT4* co-express with genes involved in photoperiod response (*CKA1, CKA2, LHY,* and *ADG1*).

In the negative GCN, a vernalization-associated gene *AGAMOUS-like 19* (*AGL19*) negatively correlates with photoperiodic and meristem identity genes (Additional file 3: Figure S3).

### Differential expression of the “flowering” genes

For differential expression of selected candidate genes that are involved in flowering, we combined the results of the in silico analyses of 74 genes of interest (Additional file 5: Table S2) with qRT-PCR analysis of 15 putative genes, annotated for their involvement in various flowering pathways.

**Fig. 6:**
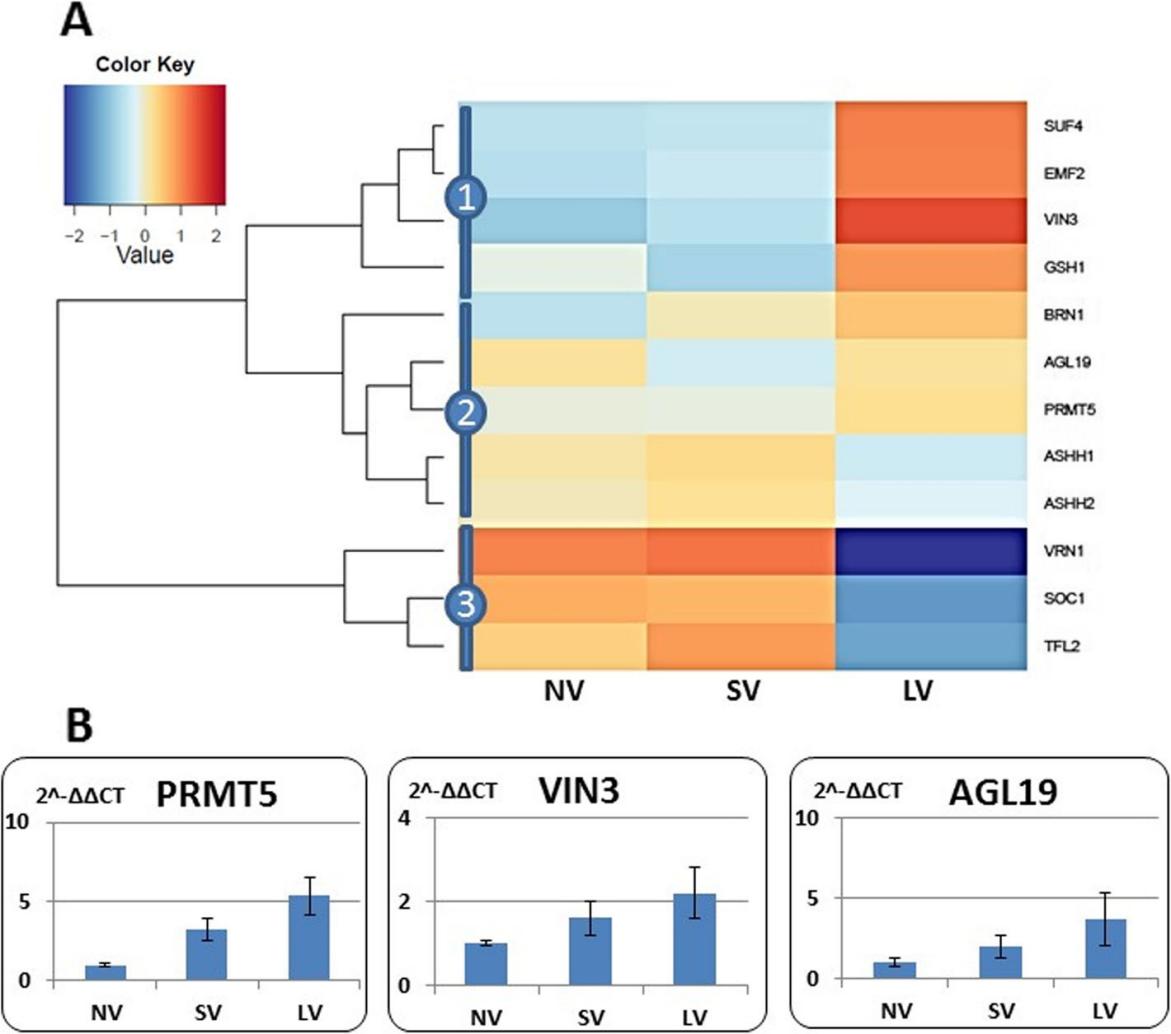
Effect of vernalization treatments on the expression of the genes associated with vernalization pathway. **a** in silico analysis of 12 vernalization-annotated genes, grouped into three clusters; The expression values (FPKM; average of the replications) were log2-transformed. **b** qRT-PCR validation of PRMT5, VIN3, and AGL19 relative expression

The genes whose expression is associated with vernalization are clustered in three large groups (Fig. 6a). One that contains homologs of *SUF4, VIN3,* and *EMF2,* shows low expression in NV and SV tissues but a strong expression in LV plants. A second cluster consists of genes slightly upregulated in LV, and the third contains *VRN1*, *SOC1, TFL2* whose over-representation is clear in NV and SV plants, but they are downregulated in LV plants. qRT-PCR expression analysis of three genes assigned to this group, *PRMT5, VIN3,* and *AGL19*, show consistent over-representation in vernalized bulbs (Fig. 6b).

**Fig. 7:**
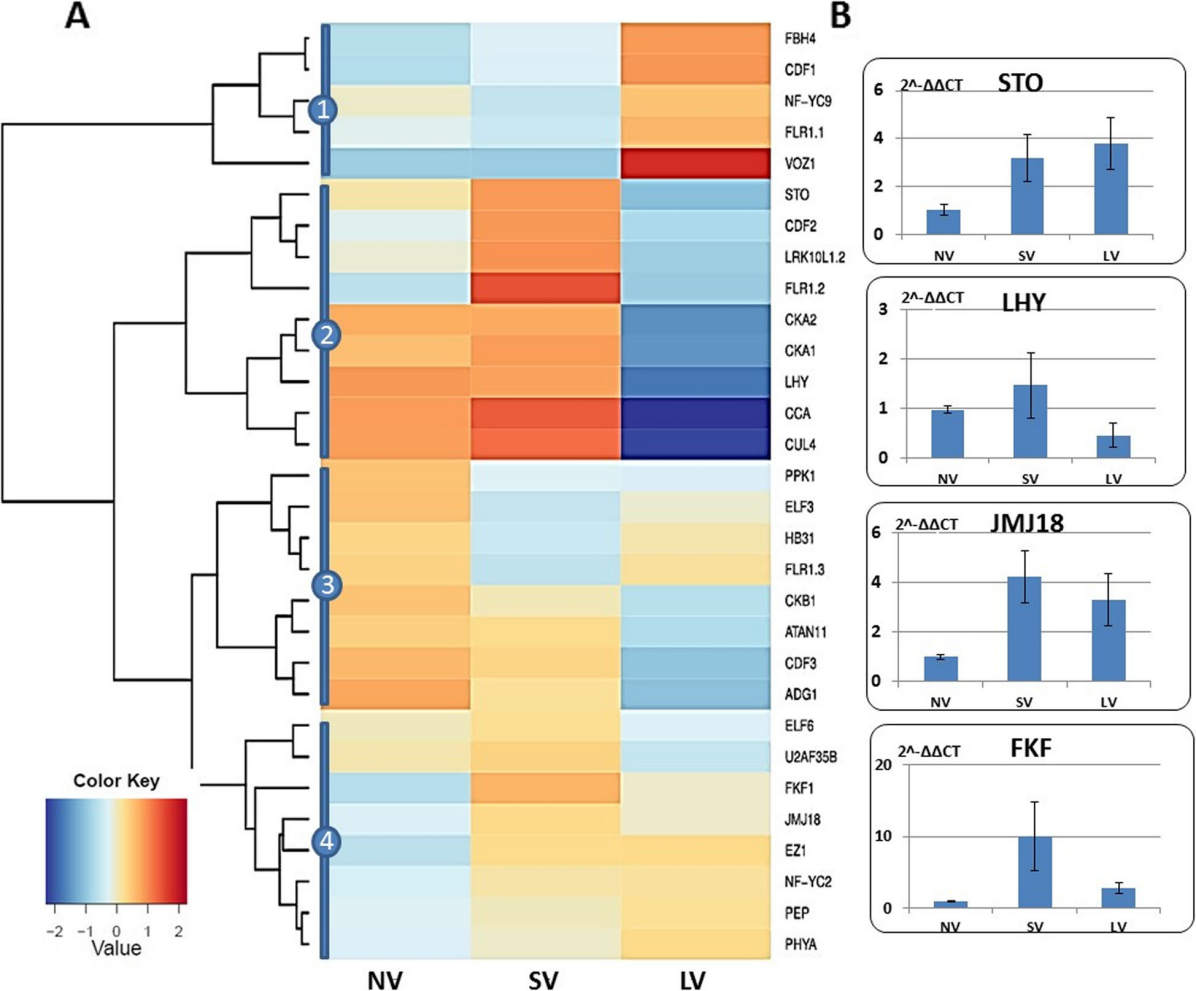
Effect of vernalization treatments on the expression of the genes associated with photoperiodic pathway. **a** in silico analysis of 30 annotated genes (Additional file 4: Table S1), grouped into four clusters; The expression values (FPKM; average of the replications) were log2-transformed. **b** a relative expression of four genes, as validated by qRT-PCR

In silico analyses of genes associated with the photoperiodic pathway generated four clusters of the differential expression (Fig. 7a). One cluster includes genes over-represented in LV plants, with exceedingly high over-expression of *VOZ1*, known for its involvement in positive regulation of long-day photoperiodism, cold acclimation and flowering. Another cluster consists of up-regulated genes, especially *FLR1.2*, in SV plants, while LV resulted in down-regulation of these genes. The same cluster includes also a group of circadian rhythm genes (*CKA1, CKA2, LHY, CCA, CUL4*) that are over-represented following both NV and SV treatments and down-regulated after long vernalization. The other two clusters show moderate expression differences between treatments. Some are slightly down-regulated after prolonged vernalization (e.g., *CKB1, ATAN11, CDF3, ADG1*) (Fig. 7a). qRT-PCR expression analyses of four specific genes *STO, LHY, JMJ18,* and *FKF,* show their overexpression mainly in SV, and *STO* and *JMJ18* responded similarly also to LV (Fig. 7b).

**Fig. 8:**
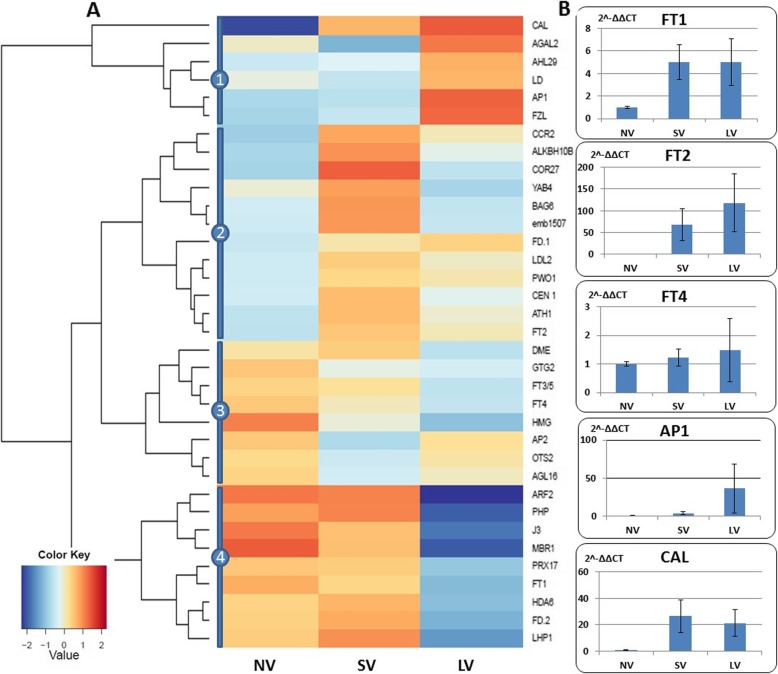
Effect of vernalization treatments on the expression of genes associated with meristem identity. **a** in silico analysis of 34 annotated genes (Additional file 4: Table S1), grouped into four clusters by their expression pattern; The expression values (FPKM; average of the replications) were log2-transformed. **b** a relative expression of four meristem-identity genes, as validated by qRT-PCR

Microscopic examination of apical meristems on the day storage treatment ended clearly show a vegetative appearence, irrespective of treatment [data not shown]. Transcriptome analyses, however, indicates high expression of numerous genes, annotated as active in meristem identity. The heatmap consists of at least four distinct clusters of genes (Fig. 8a). Most DEGs associated with meristem transition from vegetative to the reproductive state were down-regulated after LV, but *AGAL2, AHL29, LD* and *FZL* in one cluster were over-represented after LV treatment. Another cluster consisted of down-regulated genes in NV and LV plants, while SV resulted in a considerable up-regulation. A third cluster represents minor up-regulation at NV, while the fourth cluster contains over-represented genes in NV and SV bulbs (*ARF2, MBR1, J3, FT1, FD2*) that are remarkably down-regulated after long cold storage (Fig. 8a). It should be noted that RT-qPCR method has higher variance in comparison with RNAseq results and, therefore, the expression results obtained by these methods might vary. The qRT-PCR measurements of *FT1* and *FT4* expression were not always consistent with the in silico results, but the relative expression of *CAL* and *AP1* confirmed the calculated trends (Fig. 8b).

### KEGG analysis of the circadian rhythm genes

**Fig. 9:**
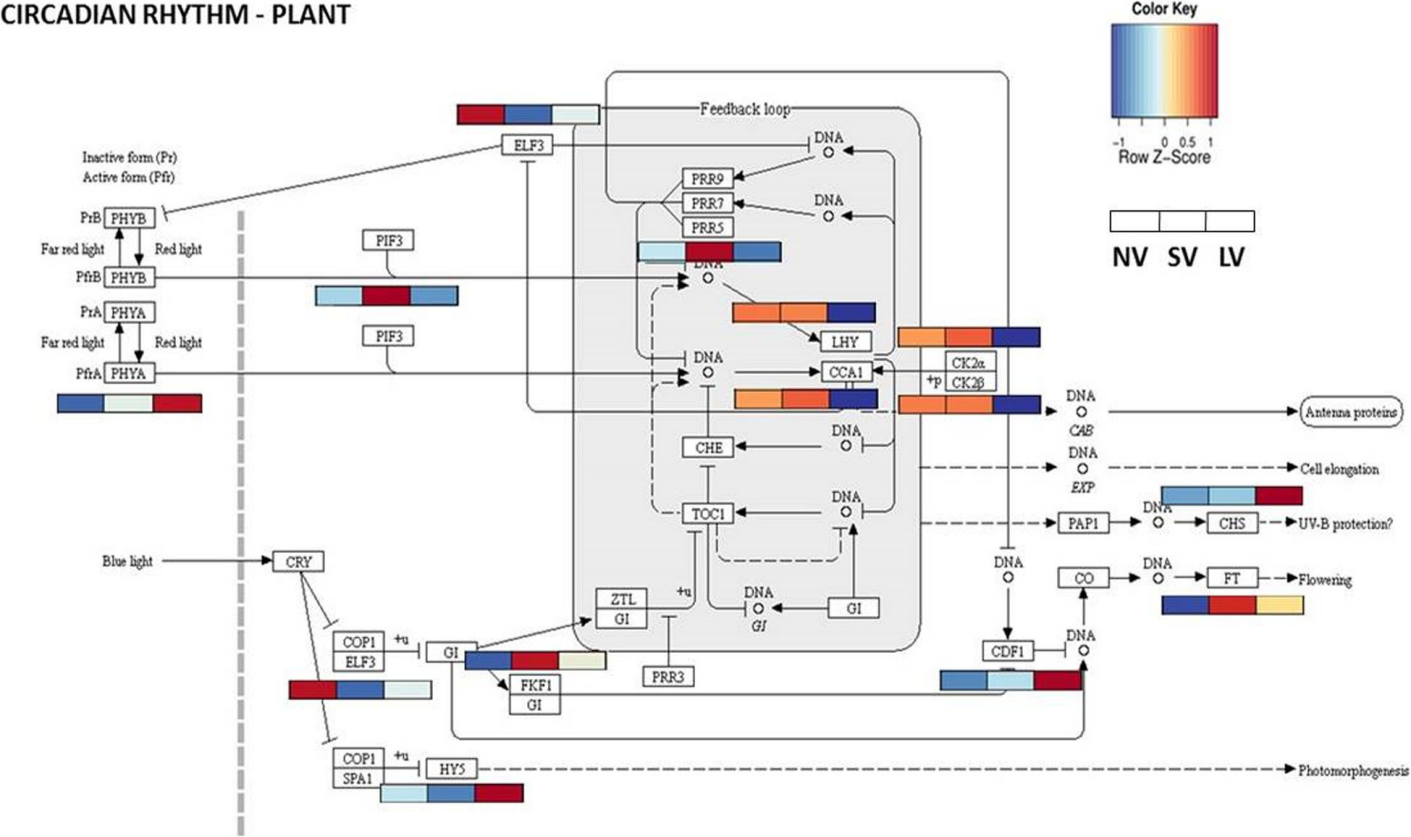
In silico analyses of the effect of vernalization treatments on the specific DEGs involved in the circadian rhythm. The KEGG algorithm employed the pathway map ko04712. Relative expression is quantified by comparing transcriptome data from #87 garlic. Differentially expressed genes are highlighted according to the expression values (FPKM; average of the replications) which were log2-transformed

The endogenous circadian oscillator provides plants with information on the daily changes and thereby controls a number of developmental processes. The analysis of the specific DEGs involved in the circadian rhythm (ko04712) using KEGG algorithm, reveals that the expression of at least half of the number of genes presented in the circadian rhythm is affected by temperature (Fig. 9). Thus, despite the complete darkness in storage, morning components *LATE ELONGATED HYPOCOTYL* (*LHY*) and *CIRCADIAN CLOCK ASSOCIATED1 (CCA1)* showed relatively high expression at the end of NV and SV and low expression on completion of the LV treatment.

Flowering promoting genes *FLAVIN-BINDING KELCH REPEAT F-box* 1(FKF1) and *FT* were over-expressed in SV compared to NV and LV treatments. The expression of *PHYTOCHROM B (PHYB)* was not significantly affected by treatments, while strong expression of *PHYTOCHROM INTERACTING FACTOR 3 (PIF3)* was evident in LV plants.

## Discussion

### Long vernalization activates meristem transition in garlic

Many geophyte species, including alliaceous crops, evolved in winter-cold climates and require vernalization for successful completion of their annual development and flowering [34, 39]. In onion and garlic, photoperiod is also considered the significant environmental cue for the transition from active growth to dormancy period [58, 70]. Our data support the findings from Ben Michael et al. [7] that long vernalization alone satisfies the needs for apical meristem transition - the first and obligatory step for flowering and bulbing in garlic. Here we studied the effect of cold treatment on the genetic interplay involved in meristem transition to active growth and flowering.

Our results show the dominant environmental cue of low temperature for triggering meristem transition (Fig. 1), while photoperiod might play only a regulatory role at the later stages of plant development [38]. It should be noted, that in onion low temperatures promote flowering and long photoperiod induces bulbing [70]. In garlic, however, cold satisfied the requirements for both processes [7]. Therefore, the molecular control of meristem transition and further plant development might vary between *A. cepa* and *A. sativum*.

### Epigenetics is involved in meristem shifting in garlic

Transcriptome analysis revealed significant modifications in epigenetic and chromatin-based processes in the apical meristems in association with pre-planting storage conditions (Fig. 3). It is widely accepted that methylation plays a central role in adaptation to environmental stress and in plants’ “winter memory” [28, 42]. Our study shows significant methylation activity in all comparisons in both, vernalized and non-vernalized bulbs, and these pathways should be further investigated.

### Vernalization in dark triggers light reaction and photoperiod-associated genes

The meristem transcriptional changes coded for major modifications in two important metabolic routes: (1) dormancy release and initiation of active growth, and (2) meristem transition from vegetative to reproductive state, with the consequent end of apical dominance and outgrowth of axillary buds. Previous physiological and molecular studies suggested that the temperature-regulated dormancy release is associated with hormone transport, and carbon, peptide and flavonoids’ metabolism [59, 63, 66, 93]. We now provide evidence that for the same physiological change, at the transcription level, numerous genes are involved in cell division, differentiation, and growth (Figs. 2b and 3). Garlic meristems reacted to vernalization by intense re-organization of a large range of cellular components and metabolic processes (Fig. 4). In dark-stored vernalized meristems, we found a large number of GO terms associated with light reaction and photosynthesis. We thus conclude that the tagged genes annotated as involved in “photosynthetic activity” are basically regulated here by low temperature. Similar results were obtained for the evergreen orchardgrass, where vernalization and photoperiod genes were involved and co-expressed during meristem transition [20]. Leeggangers et al. [46] reported that GO terms of photomorphogenesis, photoperiodism, response to light stimulus, and circadian rhythm were upregulated in dormant tulip bulbs during floral induction by high summer temperatures. They offered the explanation that the aboveground drying leaves may receive light or photoperiodic signals. These cues are then passed on to the meristem with the consequent flower induction effect. In our experiments, however, leaves and roots were all removed prior to the bulbs storage in the dark, hence light induction of photoperiodic genes was unlikely. We thus conclude that upregulation of the above genes is exclusively cold dependent.

### Gene co-expressing during meristem transition

In plants, meristem transition from the vegetative to reproductive state is coded by a multifaceted genetic network involving photoperiod, gibberellins, vernalization and autonomous signaling modules [2, 21, 61, 84]. The reception of photoperiod cue provides reliable information on seasonal changes and is thus well-conserved [83], whereas vernalization requirements and their genetic make-up vary considerably among species. The latter variation stems from the fact that acquisition of winter memory evolved independently in several plant taxa for better adaptation to local climate cycles [10]. Thus, considerable differences exist in vernalization perception between *Arabidopsis* and related species (Brassicaceae; [60]), cereals (Poaceae; [74]), Rosaceae [41] and others. We employed garlic transcriptome catalog [37] in a comparative study with a large number of genes and pathways. These analyses of flowering genes revealed a few modules in gene co-expression network (Fig. 5). While garlic transcripts annotated as vernalization genes took only minor parts in this network, they were centrally located. Vernalization genes co-expressed with numerous genes associated with light reaction and with those involved in the photoperiodic control of flowering (Fig. 5). Therefore, we propose that the molecular cascade of vernalization includes, to some extent, genes coding for the photoperiodic pathway. The latter are homologs to the *Arabidopsis STO* (major role in response to light and photoperiodism, [50]), *LHY* (circadian rhythm and photoperiodism, [22]), *FKF1* (regulation of circadian clock-dependent transition to flowering time; [88]), *VOZ1* (promotion flowering downstream of phytochrome B (phyB), [96]) and *CKA1* and *CKA2* (circadian rhythm, photomorphogenesis, [55]).

Treatment effects on differential expression of vernalization and photoperiod genes were evident (Fig. 8). A large number of those genes were downregulated in LV plants (Figs. 6 and 7), thus indicating that, on a molecular level, a transition to the reproductive stage had already occurred by the end of the LV treatment. Indeed, the differential expression of the meristem-identity genes supports this assumption (Fig. 8).

Flowering signal integrators, such as the *Arabidopsis TFL1*, play an important role as a hub connecting between the photoperiod and temperature coding genes during meristem transition [85]. In strawberry, *FvTFL1* integrates the photoperiod and temperature signals to repress flowering [73]. In garlic, gene co-expression analysis pointed out a few genes with a putative key role in the flowering process. Hence, *SUF4* links between the three modules that positively co-express with numerous flowering genes (Fig. 5), while a vernalization-associated gene *AGL19* negatively correlates with photoperiodic and meristem identity genes and seems to have a considerable repressing effect on these genes (Additional file 3: Figure S3). Therefore, *SUF4* and *AGL19* are likely to serve as floral integrators in garlic (and other geophytes), but further research is needed to determine their actual role in floral initiation.

The onset of flower development in *Arabidopsis* is controlled by the interplay between *LEAFY (LFY), APETALA1 (AP1)* and its paralog *CAULIFLOWER (CAL)* [24]. We have already shown that garlic homolog of *LFY* (*gaLFY*) acts as a key-gene in the floral transition, as well as in inflorescence and flower differentiation [77]. Our present findings show only a weak expression of *CAL* and *AP1* in non-vernalized plants and a clear upregulation following vernalization (Fig. 9). We thus propose that the latter genes interact with *gaLFY* during floral transition in garlic.

### *FT* genes are involved in meristem transition

In addition to the important and conserved role of *FT* as the ‘florigen’, other genes of this family serve in many plant species as “master” regulators of an array of functions. In geophytes, they are also involved in bulbing/tuberization [1, 45] and are associated with vernalization, photoperiod, and heat response [47, 65]. In garlic, six *FT* homologs were identified [37], including *AsFT2*, a homolog of onion ‘florigen’, whose expression well correlates with flowering initiation in garlic [45, 76].

Flowering and bulbing in onion, bunching onion (*A. fistulosum*) and many other geophytes are two competitive processes on both physiological and molecular levels [11, 17, 45, 70]. Our present results show that similar to onion, in garlic low temperatures affect down-regulation of *AsFT4* and up-regulation of *AsFT2*. Differences in *FT* expression between short- and long-day onion varieties were already shown [56, 57]. Since in vernalized garlic bulbing is also possible under short day, *FT* genes in this species can express and function in different manner, and further functional characterization of this family in various garlic genotypes will help to understand their role in florogenesis and bulbing.

### Circadian clock acts as integrator between temperature and meristem transition

Plant circadian clock, an endogenous timing system based on a cellular self-sustained oscillation [62], can be adjusted by light cycles and temperature, and may thus serve as an integration point for the two major environment cues [13, 23]. Indeed, our data show a strong expression of genes associated with the internal clock cycle, e.g., *CKA1/2, LHY, ELF3, PIF3* in vernalized garlic (Fig. 9), as previously demonstrated in chestnut and other plant species [23, 30]. The involvement of circadian rhythm in low-temperature induced florogenesis was also confirmed in lily [52], orchard grass (*Dactylis glomerata*; [20]), radish (*Raphanus sativus*; [53, 64]), Chinese cabbage (*Brassica rapa ssp. pekinensis*; [33]), as well as in bud dormancy break in trees [51, 68].

**Fig. 10:**
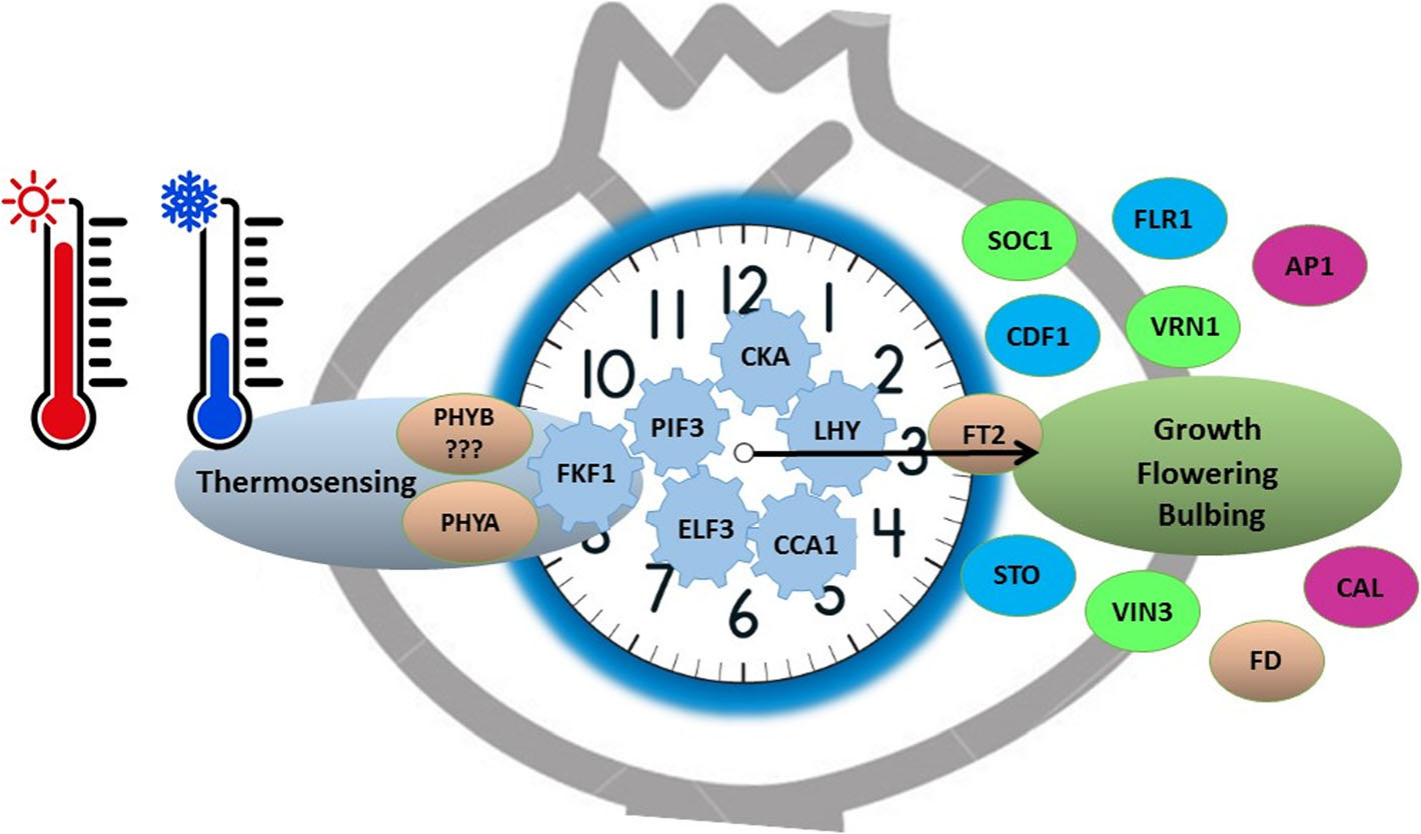
The proposed scheme of integration between vernalization, circadian rhythms and photoperiod-associated genes in garlic. The circadian clock senses and integrates the low-temperature signal and induces the expression of genes associated with vernalization, photoperiod and meristem transition

We argue that during dark cold storage of bulbs, the circadian clock integrates the low-temperature signal and consequently induces the expression of genes associated with vernalization, photoperiod and meristem transition (Fig. 10). Indeed, recent reports demonstrated that *Phytochrome B* (*PHYB),* a major light sensor of the circadian rhythm, acts as thermosensor [32, 48, 69], while *EARLY FLOWERING 3* (*ELF3*) transmits thermal information [19]. Similarly, in garlic, homolog of *PhyB* may act as thermosensor, conveying temperatures information to the circadian clock via an interface with *Phytochrome Interacting Factors* (*PIF*s) [78]. Moreover, cold induced the differential expression of at least 14 circadian clock genes, tightly connected to the core of the photoperiodic pathway (e.g.*, LHY, CCA1, FKF1, CDF1,* and *PRRs*; [22]) (Fig. 9). We conclude that molecular control of vernalization in garlic integrates into the reserved photoperiodic pathway in an upstream point, possibly by the same receptors.

## Conclusions

Long dark cold exposure of bulbs is a major cue for flowering and bulbing, and its interactions with the genetic makeup of the individual plant dictate the phenotypic expression during growth stage. Low temperatures stimulate large cascades of molecular mechanisms in garlic, and a variety of flowering mechanisms, including photoperiodic pathway, operate together via circadian clock for the benefit of meristem transition, annual life cycle and viable reproduction results.

## Methods

### Plant materials, storage treatments, and growth conditions

Flowering hardneck garlic #87, selected in 2004 from a segregating seedling population and then clonally propagated at the Agricultural Research Organization (ARO), The Volcani Center, Israel, was used in our experiments (Additional file 1: Figure S1). Plants were grown in 2015–2016 at the Experimental Farm, the Robert H. Smith Faculty of Agriculture, Food and Environment, The Hebrew University of Jerusalem, Rehovot, Israel (latitude 31.9°N).

In July–August 2015, freshly harvested bulbs were stored under ambient conditions (22–35 °C), in a roofed open-shed. The experiment was designed to ensure that all bulbs are planted at the same time to avoid seasonal variations*.*In August healthy intact uniform sized bulbs were placed in a temperature-controlled dark chamber at 4 °C for 12 weeks (LV, long vernalization), and in October for 4 weeks (SV, short vernalization). Control bulbs (NV, no vernalization) were kept in the original shed where ambient temperatures ranged between 20 and 30 °C. On November 11, 2015, when all cold treatments ended, uniform-sized cloves from healthy intact bulbs were sampled for RNA extraction and concomitantly intact cloves were planted in the soil, in a 30% shaded screenhouse, in triplicate randomized block design, 30 cloves per replication, at a 50 plants/m^2^ density. Indoors air temperature records and photoperiod for the entire growth period are presented in Additional file 2: Figure S2. Standard agriculture practices were applied throughout, yet for each treatment, fertigation [“Shefer” liquid fertilizer (N:P:K = 59:35:94 g L-^1^), Dshanim, Israel] discontinued when the aboveground parts died back, in May–June 2016, and bulbs were selectively harvested upon maturation. Twenty intact growing plants per replication (60 plants/treatment) served for phenology and morphology studies. On full bloom, floral buds and flowers were detached from the receptacle and counted in ten inflorescences per treatment. After harvest, foliage leaves and floral stalk were detached from the bulb, for fresh and dry weight measurements. The weight and structure of cured bulbs were recorded 3 weeks after harvest. Data on plant development were subjected to one-way analysis of variance (ANOVA) with the Tukey-Kramer test using JMP software [31].

### Tissues sampling for RNA/DNA analyses and extraction procedures

When storage treatments ended, three biological replications of apical buds were sampled and immediately dipped in liquid nitrogen before storage at − 80 °C. Total RNA was extracted using the CTAB protocol [14], and extract quality was assessed using the Agilent 2100 Bioanalyzer. Only extract with a minimum RNA integrated number value of seven were used in the following studies.

### Transcriptome assembly

Library preparation and sequencing were performed by the Genome Center, Life Sciences and Engineering, Technion, Israel. Six libraries of paired-end RNA-seq of 100 nucleotides, were prepared for analysis using Illumina Hiseq 2500 and Trueseq protocols. Raw reads were subjected to a cleaning procedure with FASTX Toolkit (http://hannonlab.cshl.edu/fastx_toolkit/index.html, version 0.0.13.2) as follows:

1. trimming read-end nucleotides with quality scores < 30 using fastq_quality_trimmer;
2. read pairs were discarded if either one had less than 70% base pairs with quality score ≤ 30 using fastq_quality_filter.

Following processing and cleaning, a total of approximately 230 million cleaned paired-end reads were assembled de novo*,* using Trinity software (Trinityrnaseq version v2.3.2, [25]) with default parameters by Trimmomatic option.

The resulting data were compiled with previously published transcriptome catalog of fertile garlic [37]. The new transcriptome catalog now contains a total of 112,388 trinity genes with an average length of 1.178 bp and N50 of 1812 bp. The data have been deposited in the NCBI Sequence Read Archive (SRA) database as a bioproject PRJNA566287.

### Abundance estimation and differential expression analysis

The cleaned reads from each library were aligned with the newly assembled transcriptome, using the Bowtie2 aligner [43]. The abundance estimation was calculated using the Trinity protocol [27] and the Expectation-Maximization method (RSEM). The normalized expression was calculated by TMM (trimmed mean of M values) normalization and by FPKM (fragments per kilobase of transcript per million mapped reads).

Comparisons between each pair of samples and differential expression analyses of the sequence count data were performed by Bioconductor edgeR package in the R environment [75]. Differential expression was defined as a value larger than the two-fold difference in transcription expression with a false discovery-corrected statistical significance below 0.05. The differentially expressed transcripts that had a minimum of one pairwise comparison between treatments were examined, using cluster analysis. Based on the means of two replications, hierarchical clustering of transcripts and samples was performed and clusters were extracted using R scripts.

### Functional annotations

The assembled transcriptome was used for a search of the NCBI non-redundant (nr) protein database, employing the DIAMOND software [12]. Homologous sequences were identified within the Swiss-Prot database using the BLASTX tool [3] at *E*-value threshold of 10^− 5^. The results were exported to Blast2GO version 4.0 [15] for gene ontology (GO) assignments. The KAAS tool (KEGG Automatic Annotation Server; http://www.genome.jp/tools/kaas/) was used for KEGG orthology and KEGG pathway assignments. Gene ontology enrichment analyses were carried out using Blast2GO [15] software based on Fisher’s Exact Test [92] with multiple testing correction of false discovery rates (FDR; [6]). The threshold was set as FDR with corrected *P*-value smaller than 0.05. The ReviGO web server was used for visualization of the GO terms in a semantic similarity-based scatterplot (http://revigo.irb.hr; [87]).

A gene co-expression network (GCN) was constructed based on the Pearson correlation (using R scripts). Networks were plotted using Cytoscape 3.3.0 ([81]; http://www.cytoscape.org).

### Real-time quantitative PCR validation

For gene expression validation, 15 genes were selected upon their putative function in flower induction and meristem transition. The RNA samples employed for the construction of libraries and transcriptome analyses were further analyzed. Two micrograms RNA samples from each of the total RNA obtained from three samples/storage treatment, served for the synthesis of cDNA using the High-Capacity Reverse Transcription Kit (Applied Biosystems, Foster City, CA). Primers were designed using primer3.0 web-tool (Additional file 4: Table S1). Amplicon identities were validated by sequencing with Sanger sequencing (Life Sciences Core Facilities, Weizmann Institute of Science, Israel).

Gene expression was validated using the Fluidigm® system. The cDNA samples along with non-template control (NTC) were analyzed by Biological Services, Weizmann Institute for Science, Israel. Each of the three technical repetitions per sample was analyzed twice for all sets of primers by using the 48.48 Dynamic Array™ IFCs, the BioMark™ HD System (Fluidigm, South San Francisco, CA) and GE Fast PCR + Melt v2 Protocol (https://www.fluidigm.com/documents). Data analysis was performed using Fluidigm Real-time PCR software. Garlic homologs of actin (AY821677) and tubulin (AY148156) served as reference genes for data normalization and calculation of relative amounts of mRNA in the studied samples. All sequences were deposited in the garlic transcriptome catalog (NCBI bioproject PRJNA566287). The expression was quantified by the ∆∆CT method [54] and presented in a log scale (2^-ΔΔCT^).

## Supplementary information

**Additional file 1: Figure S1.** Flowchart depicting the experimental design, including pre-planting treatments, morpho-physiological studies, transcriptome analyses and validation of candidate genes in #87 garlic.

**Additional file 2: Figure S2.** Day length and mean daily temperatures from October 2015 to September 2016, in Rehovot, Israel.

**Additional file 3: Figure S3.** Negative co-expression of garlic genes associated with vernalization (green), photoperiod (blue) pathway and meristem transition (purple). Data from three vernalization treatments were analyzed using the network-drawing software Cytoscape [80]. Pearson correlation value higher than 0.9.

**Additional file 4: Table S1.** List of primers designed for qRT-PCR validation. Garlic homologs of actin (AY821677), tubulin (AY148156), and NADH (TRINITY_DN98625_c0_g3) served as reference genes for data normalization and calculation of relative amounts of mRNA in the studied samples.

**Additional file 5: Table S2.** List of 74 genes associated with meristem transition, photoperiodic pathway and vernalization, their relevant GO terms, and short description according to UniProt database and literature survey.

## Data Availability

The datasets generated during the current study are submitted to the NCBI repository, bioproject PRJNA566287.

## Abbreviations

DAP: Days after planting
LV: Long vernalization
NV: No vernalization
SV: Short vernalization
