# Supplementary figures and images for: Crosstalk in the darkness: bulb vernalization activates meristem transition via circadian rhythm and photoperiodic pathway
Source: BMC Plant Biol. 2020 Feb 17;20:77. doi: 10.1186/s12870-020-2269-x (PMC7027078; doi:10.1186/s12870-020-2269-x)

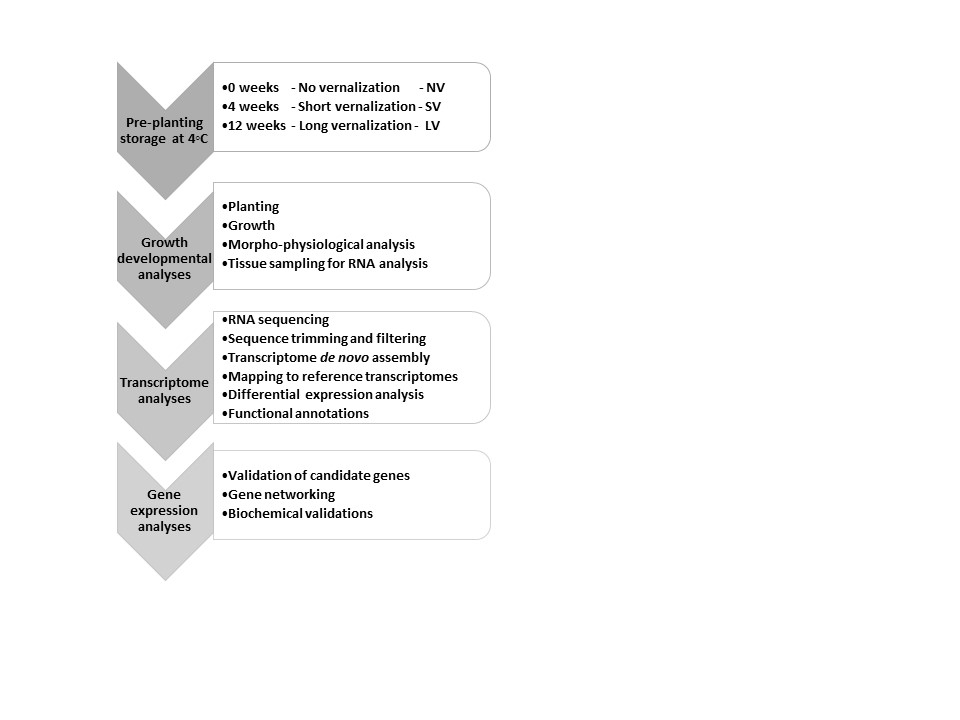

Supplement: Supplementary file 1 — Additional file 1: Figure S1. Flowchart depicting the experimental design, including pre-planting treatments, morpho-physiological studies, transcriptome analyses and validation of candidate genes in #87 garlic. [file 12870_2020_2269_MOESM1_ESM.tif]

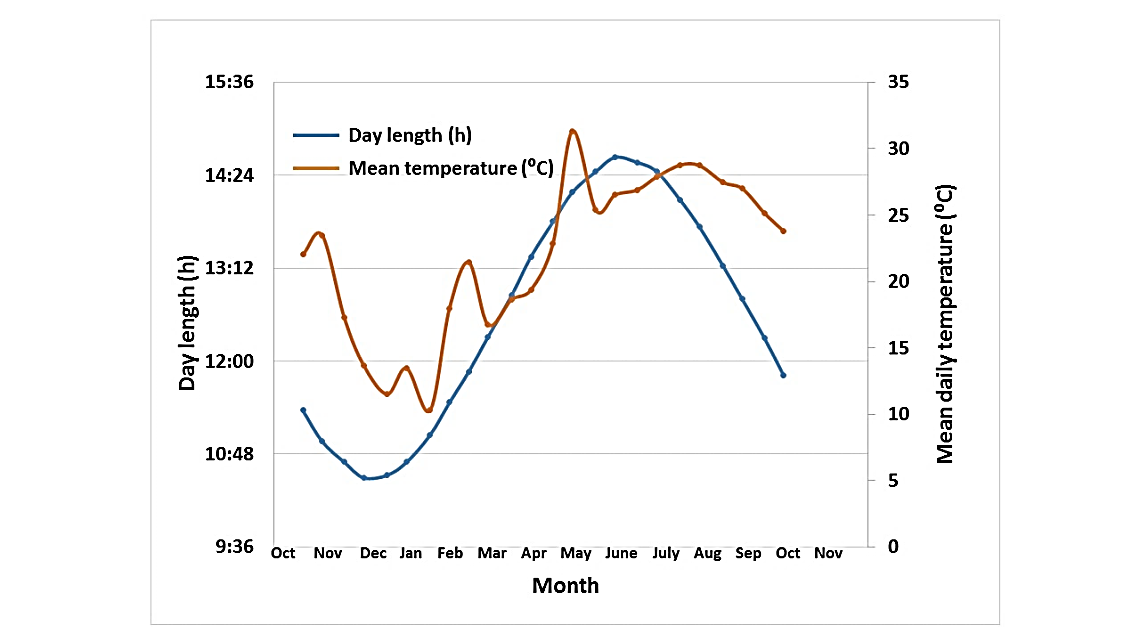

Supplement: Supplementary file 2 — Additional file 2: Figure S2. Day length and mean daily temperatures from October 2015 to September 2016, in Rehovot, Israel. [file 12870_2020_2269_MOESM2_ESM.tif]
