# Supplementary material for: Crosstalk in the darkness: bulb vernalization activates meristem transition via circadian rhythm and photoperiodic pathway
Source: BMC Plant Biol. 2020 Feb 17;20:77. doi: 10.1186/s12870-020-2269-x (PMC7027078; doi:10.1186/s12870-020-2269-x)
Supplement: Supplementary file 3 — Additional file 3: Figure S3. Negative co-expression of garlic genes associated with vernalization (green), photoperiod (blue) pathway and meristem transition (purple). Data from three vernalization treatments were analyzed using the network-drawing software Cytoscape [80]. Pearson correlation value higher than 0.9. [file 12870_2020_2269_MOESM3_ESM.docx]

**
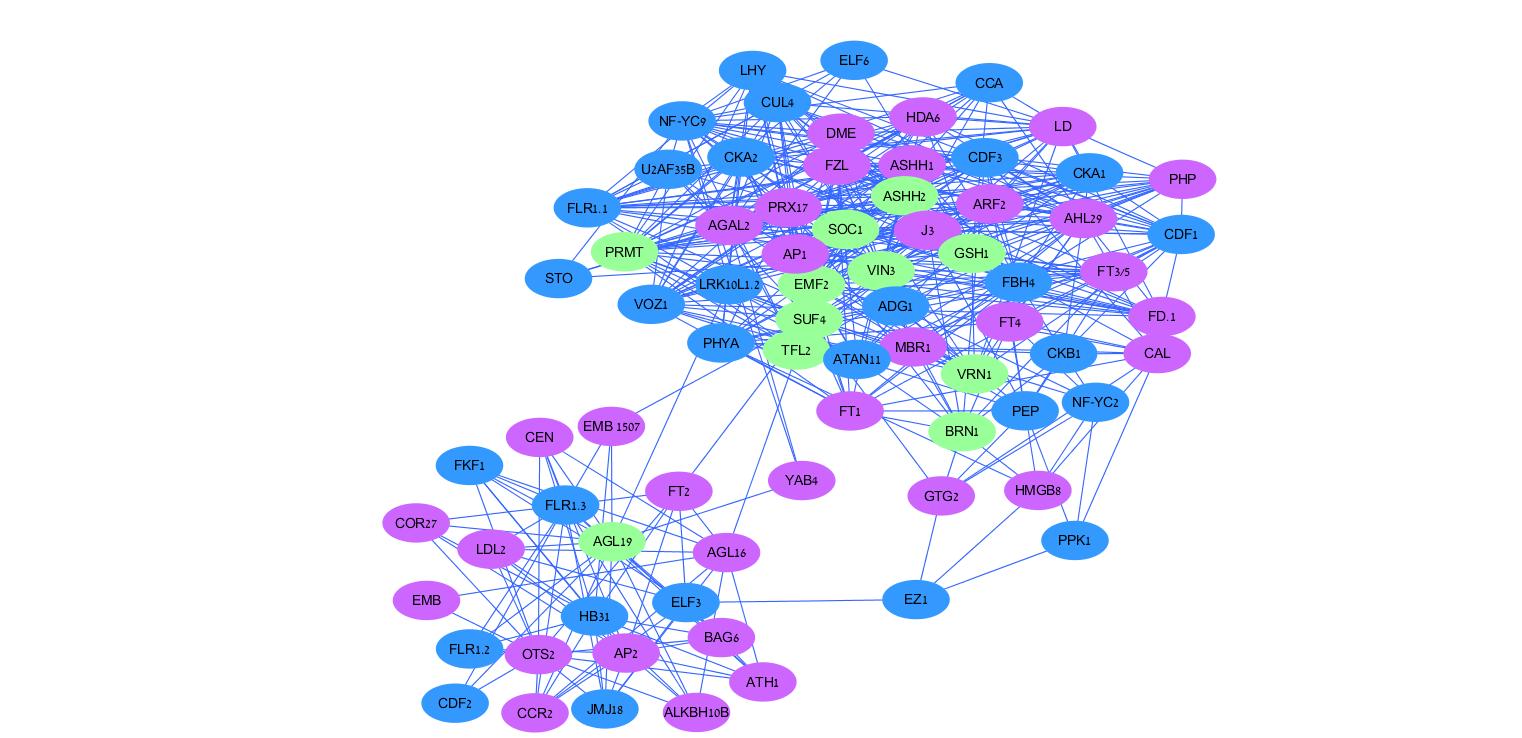
**

Fig. S3. Negative co-expression of garlic genes associated with vernalization (green), photoperiod (blue) pathway and meristem transition (purple). Data from three vernalization treatments were analyzed using the network‐drawing software Cytoscape (Shannon et al., 2003). Pearson correlation value higher than 0.9.
