# Supplementary material for: Crosstalk in the darkness: bulb vernalization activates meristem transition via circadian rhythm and photoperiodic pathway
Source: BMC Plant Biol. 2020 Feb 17;20:77. doi: 10.1186/s12870-020-2269-x (PMC7027078; doi:10.1186/s12870-020-2269-x)
Supplement: Supplementary file 4 — Additional file 4: Table S1. List of primers designed for qRT-PCR validation. Garlic homologs of actin (AY821677), tubulin (AY148156), and NADH (TRINITY_DN98625_c0_g3) served as reference genes for data normalization and calculation of relative amounts of mRNA in the studied samples. [file 12870_2020_2269_MOESM4_ESM.docx]

Table S1. List of primers designed for qRT-PCR validation. Garlic homologs of actin (AY821677), tubulin (AY148156), and NADH served as reference genes for data normalization and calculation of relative amounts of mRNA in the studied samples.

| **Gene name** | **Trinity ID** | **Forward** | **Reverse** | **Position** |
| --- | --- | --- | --- | --- |
| *PRMT5* | TRINITY_DN100857_c2_g3 | CCATTCGCATCATCTGATTT | GCTGATTTTTCCAACAACATGA | 151-219 |
| *VIN3* | TRINITY_DN94103_c1_g11 | GGAAGAACAGCCTTCGTTTC | CACCAAAAGCGTCGAACTAA | 424-493 |
| *AGL19* | TRINITY_DN106352_c1_g1 | GCCCACTGTCTTCAATTGGT | GCGGGAAGAATGAACCAGAT | 1280-1398 |
| *STO* | TRINITY_DN109209_c1_g2 | GTGCATCCATCGTTCCTCTT | ACAAGGAGGCAGACTGAAGT | 62-149 |
| *LHY* | TRINITY_DN92168_c1_g5 | GAAGTTGGGATGCCGAAGTC | CCATCGAGCAACACCAAAGT | 10-107 |
| *JMJ18* | TRINITY_DN94553_c0_g4 | AGAGCACCCGAATGAGATGT | GAACTGCAGCGAGGAAATGT | 2643-2772 |
| *FKF1* | TRINITY_DN70116_c0_g1 | AGCTCACCCAAAACGAACAC | TAGGGCTCGACTGCTTGATT | 873-967 |
| *FT1* | TRINITY_DN104463_c2_g3 | TCGCAAACCCTCTTCCTCTT | TTGAAGCTCCGCAATCACAA | 87-226 |
| *FT2* | TRINITY_DN87300_c2_g1 | TGTATAAGTTCTCCTCCCACCT | CAGAAGTATACAACTTGGGTTCTC | 197-277 |
| *FT4* | TRINITY_DN105192_c3_g1 | GCTCACATTTCCTGCGACTT | AAATTGGTGACGCTGCCTTT | 1899-1959 |
| *AP1* | TRINITY_DN87548_c0_g1 | AGCGCTCAAGAATCCTATCCA | TTCTTTGCGACGCTGATGTT | 937-1035 |
| *CAL* | TRINITY_DN106685_c1_g2 | AGACCGACTTCAGCATCACA | AGGAGGAGCGGATTGTTGAA | 466-530 |
| *FLX3* | TRINITY_DN89789_c0_g2 | GCTGAGCAAATGGAGCAGAA | CTTGCATTCTCCGCCTTCAA | 262-350 |
| *FPA* | TRINITY_DN102611_c2_g7 | TCAATCTTCCCAAACTGCAA | TTAGCAAAGCTGTCTCTGAAGAA | 34-94 |
| *FLK* | TRINITY_DN97071_c1_g9 | AGCATTTGACGATGGCTGTT | ACCTGCCGAGATTCCTTCAT | 714-816 |
| *ACT* | TRINITY_DN102589_c4_g8 | GAGCCACACAGTCCCGATAT | CTGCTGTGGTGGTGAATGAG | 704-840 |
| *TUB* | TRINITY_DN99629_c1_g1 | GGAGGATGCTGCAAACAACT | CAGCTTTCGGATGCGATCAA | 398-485 |
| *NADH* | TRINITY_DN98625_c0_g3 | TGCCGGCGTTGATATTCCTA | TTCTCCACCTCAACCAAGCA | 314-400 |
