# Supplementary material for: Crosstalk in the darkness: bulb vernalization activates meristem transition via circadian rhythm and photoperiodic pathway
Source: BMC Plant Biol. 2020 Feb 17;20:77. doi: 10.1186/s12870-020-2269-x (PMC7027078; doi:10.1186/s12870-020-2269-x)
Supplement: Supplementary file 5 — Additional file 5: Table S2. List of 74 genes associated with meristem transition, photoperiodic pathway and vernalization, their relevant GO terms, and short description according to UniProt database and literature survey. [file 12870_2020_2269_MOESM5_ESM.docx]

Table S2. List of 74 genes associated with meristem transition, photoperiodic pathway and vernalization, their relevant GO terms, and short description according to UniProt database and literature survey.

| **Short name** | **Full name** | **Trinity ID** | **GO values** | **Putative function** |
| --- | --- | --- | --- | --- |
| **Vernalization** | | | | |
| *SUF4* | *SUPPRESSOR OF FRIGIDA 4* | DN94780_c0_g1 | vegetative to reproductive transition of meristem | Recruits the FRI-C complex to the *FLC* promoter. Required for FRI-mediated *FLC* activation, but has no effect on the expression of *MAF1,2,3,5, UFC* and *CO*. Required to maintain high levels of *FLC* expression in later embryonic and vegetative development |
| *EMF2* | *EMBRYONIC FLOWER 2* | DN101144_c3_g3 | cell differentiation; chromatin organization; flower development;  negative regulation of flower development; regulation of gene expression by genetic imprinting | Participates in polycomb group (PcG) protein complex-mediated (probably in complex with EMF1) silencing of the flower homeotic genes *AG*, *PI*, and *AP3*, as well as of some regulatory genes such as *ABI3*, *LOV1*, and *FLC* during vegetative development, by mediating trimethylation of histone 3 lysine 27 on the *AG* chromatin (H3K27me3) |
| *FUL* | *FRUITFULL* | DN87548_c0_g1 | developmental growth involved in morphogenesis; flower development; maintenance of inflorescence meristem identity; positive regulation of flower development | Promotes early floral meristem identity in synergy with *AP1* and *CAL* |
| *VIN3* | *VERNALIZATION INSENSITIVE 3* | DN107253_c1_g2 | circadian regulation of gene expression; flower development;  positive regulation of histone H3-K9 dimethylation; regulation of short-day photoperiodism, flowering;  response to cold | May be involved in both the vernalization and photoperiod pathways by regulating gene expression. Binds preferentially to dimethylated histone H3 'Lys-9' (H3K9me2). Promotes flowering in non-inductive photoperiods (e.g. short days) through the maintenance of the epigenetically repressed state of *MAF5* via H3K9me2 and plant homeodomain / polycomb repressive complex 2 (PHD-PRC2)-dependent H3K27me3 |
| *GSH1* | *GLUTAMATE-CYSTEINE LIGASE* | DN100957_c3_g6 | vernalization-promoting flowering | GSH biosynthesis partially mimics seed vernalization treatment in *Arabidopsis thaliana*, induced a specific pattern of gene expression and promoted subsequent flowering |
| *BRN1* | *BRUNO-LIKE 1* | DN98504_c3_g1 | flower development; regulation of photoperiodism, flowering | Involved in the regulation of flowering time. Acts as repressor of the activity of *SOC1* |
| *AGL19* | *AGAMOUS-LIKE 19* | DN106352_c1_g1 | regulation of flower development; timing of transition from vegetative to reproductive phase; vernalization response | Transcription factor that promotes flowering, especially in response to vernalization by short periods of cold, in an *FLC*-independent manner. |
| *PRMT5* | *PROTEIN ARGININE N-METHYLTRANSFERASE* | DN100857_c2_g3 | histone arginine methylation; histone H4-R3 methylation; positive regulation of vernalization response; regulation of flower development | Methylates symmetrically histone H4 of the *FLC* chromatin to form H4R3me2s, which in turn suppresses *FLC* expression to induce flowering |
| *ASHH2* | *HISTONE LYSINE N-METHYLTRANSFERASE ASHH2* | DN106422_c0_g1 | histone H3-K36 methylation;  negative regulation of flower development; regulation of gene expression, epigenetic | Regulates positively *FLC* transcription to prevent early flowering transition. Required for flowering transition in response to vernalization and for the maintenance of *FLC* expression in late embryos |
| *VRN1* | *VERNALIZATION 1* | DN91020_c0_g2 | regulation of flower development;  vernalization response | Involved in the regulation of vernalization. Acts as transcriptional repressor of *FLC*, a major target of the vernalization pathway. Binds DNA in vitro in a non-sequence-specific manner |
| *SOC1* | *SUPRESSION OF CONSTANT1* | DN106352_c1_g5 | flower development; maintenance of inflorescence meristem identity; positive regulation of flower development; regulation of flower development; response to cold; vernalization response | Transcription activator active in flowering time control. May integrate signals from the photoperiod, vernalization and autonomous floral induction pathways. When associated with *AGL24*, mediates effect of gibberellins on flowering under short-day conditions, and regulates the expression of *LEAFY*, which links floral induction and floral development |
| *TFL2* | *TERMINAL FLOWER 2* | DN108112_c3_g2 | chromatin silencing; negative regulation of flower development; negative regulation of gene expression, epigenetic; photoperiodism; flowering;  vernalization response | Structural component of heterochromatin involved in gene repression, including several floral homeotic genes and *FLT* that regulates flowering time. Required for maintenance of vernalization-induced repression of *FLC* |
| **Photoperiod** | | | | |
| *FBH4* | *FLOWERING*  *BHLH 4* | DN91239_c0_g1 | photoperiodism, flowering | Controls expression of photoperiodic flowering regulator *CONSTANS* |
| *CDF1* | *CYCLING DOF FACTOR 1* | DN100145_c0_g2 | regulation of timing of transition from vegetative to reproductive phase; vegetative to reproductive phase transition of meristem | Regulates photoperiodic flowering response. Transcriptional repressor of *CONSTANS* expression. The DNA-binding ability is not modulated by *GIGANTEA* but the stability of *CDF1* is controlled by the proteasome-dependent pathway |
| *NF-YC9* | *NUCLEAR FACTOR Y, SUBUNIT C9* | DN61882_c0_g2 | [positive regulation of photomorphogenesis](https://www.ebi.ac.uk/QuickGO/term/GO:2000306) | Interacts with *REF6* to directly regulate *SOC1* transcription in response to flowering signals from photoperiod and gibberellic acid pathways |
| *FLR1* | *FLOR1* | DN82052_c0_g1; DN108911_c0_g3; DN108911_c0_g5 | long-day photoperiodism, flowering; regulation of timing of transition from vegetative to reproductive phase | Expressed in early meristem transition in response to LD, in a *SOC1 FUL* dependent manner downstream and in parallel to *FT*. *flor1* mutations delayed flowering. |
| *FKF1* | *FLAVIN-BINDING KELCH REPEAT F-BOX PROTEIN 1* | DN70116_c0_g1 | circadian rhythm; positive regulation of flower development; regulation of gene expression; response to blue light | Acts as a blue light photoreceptor that mediates light-regulated protein degradation of critical clock components by targeting them to the proteasome complex. Involved in the regulation of circadian clock-dependent processes including transition to flowering time: Forms a complex with *GIGANTEA* to regulate *CONSTANS* expression. Promotes *CO* expression during the light period of long days |
| *JMJ18* | *JUMONJI DOMAIN-CONTAINING PROTEIN 18* | DN94553_c0_g4 | [photoperiodism, flowering; histone demethylase activity](https://www.ebi.ac.uk/QuickGO/term/GO:0048573) | Involved in the control of flowering time by demethylating H3K4me3 at the *FLC* locus and repressing its expression. |
| *EZ1* | *HISTONE-LYSINE N-METHYLTRANSFERASE EZ1* | DN105190_c2_g4 | chromatin silencing; flower development; histone H3-K27 methylation; regulation of short-day photoperiodism, flowering | Polycomb group (PcG) protein. Involved in the regulation of flowering. Promotes flowering under short day conditions. Regulates the trimethylation on histone H3 'Lys-27' (H3K27me3) of the flowering regulator *Late Flowering (LF)* |
| *NF-YC2* | *NUCLEAR TRANSCRIPTION FACTOR Y SUBUNIT C-2* | DN79950_c0_g2 | flower development; negative regulation of long-day photoperiodism, flowering | Involved in the regulation of flowering time under long day conditions. Functions as repressor of flowering, independently of *Heading Date 1* and *GHD7.* Controls flowering time by negatively regulating the expression of *Early Heading Date 1* and *Heading Date 3A* (rice's FT homologue). |
| *PEP* | *PEPPER* | DN99097_c2_g1 | negative regulation of long-day photoperiodism, flowering; negative regulation of short-day photoperiodism | Antagonizes *Flowering Locus K* by positively regulating *FLC* probably at transcriptional and post-transcriptional levels, and thus acts as a negative regulator of flowering. |
| *PHYA* | *PHYTOCHROME A* | DN101672_c1_g1 | detection of visible light;  photomorphogenesis; phototropism; red light signaling pathway; response to continuous far red light stimulus by the high-irradiance response system; response to far red light;  response to very low fluency red light stimulus | Regulatory photoreceptor which exists in two forms that are reversibly interconvertible by light: the Pr form that absorbs maximally in the red region of the spectrum and the Pfr form that absorbs maximally in the far-red region. Photoconversion of Pr to Pfr induces an array of morphogenetic responses |
| *ELF6* | *EARLY FLOWERING 6* | DN104879_c1_g3 | histone H3-K9 demethylation;  negative regulation of long-day photoperiodism and short-day photoperiodism; flowering | Involved in transcriptional gene regulation. Acts as a repressor of the photoperiodic flowering pathway and of *FT* |
| *VOZ1* | *VASCULAR PLANT ONE-ZINC FINGER 1* | DN95147_c3_g2 | cold acclimation; flowering;  positive regulation of long-day photoperiodism, flowering; red, far-red light phototransduction | Promotes flowering downstream of phyB. Down-regulates *FLC* and up-regulates *FT* |
| *U2AF35B* | *SPLICING FACTOR U2AF SMALL SUBUNIT B* | DN100405_c0_g2 | [photoperiodism, flowering](https://www.ebi.ac.uk/QuickGO/term/GO:0048573) | Necessary for the splicing of pre-mRNA (by similarity). Probably active at the 3' splice sites. Mutants showed altered flowering time |
| *ATAN*  *11* | *ANTHOCYANIN11* | DN97956_c1_g3 | entrainment of circadian clock by photoperiod; photoperiodism, flowering; rhythmic process | Mutant has an early-flowering phenotype, contributed by the significant phase shift of *CO*, and, therefore, an increased expression of *FT* before dusk. |
| *CKB1* | *CASEIN KINASE II BETA CHAIN 1* | DN98075_c2_g2 | photoperiodism, flowering; protein phosphorylation; regulation of circadian rhythm | [Plays a complex role in regulating the basal catalytic activity of the alpha subunit. CK2 phosphorylates the transcription factor PIF1 after an exposure to light, resulting in a proteasome-dependent degradation of PIF1 and promotion of photomorphogenesis](https://www.uniprot.org/citations/7696877) |
| *PPK1* | *PHOTOREGULATORY PROTEIN KINASE 1* | DN104778_c0_g2 | photoperiodism, flowering;  vegetative to reproductive phase transition of meristem | Catalyze blue light in the phosphorylation process of *CRYPTOCHROME2* that regulate photoperiodic flowering |
| *ELF3* | *EARLY FLOWERING 3* | DN100005_c0_g4 | circadian clock input pathway component | Can regulate the initiation of flowering independently of *phyB* |
| *HB31* | *HOMEOBOX PROTEIN 31* | DN92249_c0_g1 | long-day photoperiodism, flowering | Putative transcription factor. Probably involved in the regulation of floral induction. The mRNA of *HB31* was not detected in the meristem prior to exposure to LDs, but after exposure to ~~three~~ LDs, it was present on the flanks of the meristem adjacent to floral primordia |
| *STO* | *SALT TOLERANCE* | DN109209_c1_g2 | photoperiodism, flowering;  vegetative to reproductive phase transition of meristem | Photoperiod and the circadian clock pathway. Can affect the key flowering time genes *FLC* and *FT/SOC1* separately. |
| *CDF2* | *CYCLING DOF FACTOR 2* | DN99931_c0_g2 | [vegetative to reproductive phase transition of meristem](https://www.ebi.ac.uk/QuickGO/term/GO:0010228) | Regulates a photoperiodic flowering response. Transcriptional repressor of *CO* expression. |
| *LRK10L1.2* | *LEAF RUST 10 DISEASE-RESISTANCE LOCUS RECEPTOR- LIKE PROTEIN KINASE-LIKE 1.2* | DN101915_c0_g1 | [photoperiodism, flowering](https://www.ebi.ac.uk/QuickGO/term/GO:0048573) | *LRK10L1.2* mutant, under the 35S promoter (35S-LRK10L1.2 No4), flowers much earlier than WT |
| *CKA1* | *CASEIN KINASE ALPHA 1* | DN96421_c0_g8 | [vegetative to reproductive phase transition of meristem](https://www.ebi.ac.uk/QuickGO/term/GO:0010228) | Mutants showed delayed flowering. Acts as circadian clock component that maintains the correct period length through phosphorylation of *CCA1*. Required for the maintenance and control of genomic stability and chromatin structure |
| *CKA2* | *CASEIN KINASE ALPHA 2* | DN96421_c0_g6 | flower development; inflorescence development; photoperiodism; regulation of circadian rhythm | Phosphorylates the transcription factor *PIF1* after an exposure to light, resulting in a proteasome-dependent degradation of PIF1 and promotion of photomorphogenesis. Acts as circadian clock component that maintains the correct period length through phosphorylation of CCA1 |
| *LHY* | *LATE ELONGATED HYPOCOTYL* | DN92168_c1_g4 | long-day photoperiodism, flowering; response to cold | Accelerates flowering in part by reducing the abundance of *SVP* and thereby antagonizing its capacity to repress *FT* expression under LD |
| *CDF3* | *CYCLIC DOF FACTOR 3* | DN73416_c0_g1 | flower development; regulation of transcription, DNA-templated | Transcription factor that binds specifically to a 5'-AA [AG] G-3' consensus core sequence (by similarity). Regulates a photoperiodic flowering response. Transcriptional repressor of *CO* expression |
| *ADG1* | *ADP GLUCOSE PYROPHOSPHORYLASE 1* | DN101795_c2_g5 | photoperiodism, flowering | Plays a role in synthesis of starch. Mutants showed late flowering in long days |
| *CUL4* | *CULLIN4* | DN94972_c1_g2 | negative regulation of photomorphogenesis; photomorphogenesis; short-day photoperiodism, flowering | Acts together with the CUL4-DDB1-COP1-SPA E3 ubiquitin-protein ligase complexes in the repression of photomorphogenesis and flowering time |
| *CCA* | *CIRCADIAN CLOCK ASSOCIATED* | DN92168_c1_g5 | circadian rhythm; long-day photoperiodism, flowering; negative regulation of circadian rhythm;  response to cold | Involved in the circadian clock and in the phytochrome regulation. Binds to the promoter regions of *TOC1* and *TCP21/CHE* to repress their transcription. Binds to the promoter regions of *CAB2A* *and CAB2B* to promote their transcription. Represses both *LHY* and itself |
| **Meristem Transition** | | | | |
| *CAL* | *CAULIFLOWER* | DN106685_c1_g2 | floral meristem determinacy;  multicellular organism development;  positive regulation of flower development | Promotes early floral meristem identity in synergy with *AP1, FUL* and *LFY* |
| *AGAL2* | *ALPHA-GALACTOSIDASE 2* | DN99136_c0_g2 | [positive regulation of flower development](https://www.ebi.ac.uk/QuickGO/term/GO:0009911) | Mutants showed delayed flowering |
| *AHL29* | *AT-HOOK MOTIF NUCLEAR-LOCALIZED PROTEIN 29* | DN89134_c0_g1 | flower development; photomorphogenesis; vegetative to reproductive phase transition of meristem | Acts redundantly with *AHL18, AHL22* and *AHL27* in the repressing regulation of flowering and regulation of the hypocotyl elongation |
| *LD* | *LUMINIDEPENDENS* | DN91738_c0_g1 | flower development; vegetative to reproductive phase transition of meristem | Expressed in regions of cell proliferation and encodes a nuclear protein that regulates *LEAFY* expression |
| *FZL* | *FZO-LIKE* | DN105670_c0_g4 | [vegetative to reproductive phase transition of meristem](https://www.ebi.ac.uk/QuickGO/term/GO:0010228) | Mutants showed delayed flowering. |
| *CCR2* | *COLD, CIRCADIAN RHYTHM, AND RNA BINDING 2* | DN81594_c0_g3 | vegetative to reproductive phase transition of meristem; response to cold | Component of the flowering autonomous pathway which promotes floral transition, at least partly by down-regulating *FLC* |
| *ALKBH10B* | *ALPA-KETOGLUTARATE- DEPENDENT DIOXYGENASE ALKB HOMOLOG 10B* | DN106655_c0_g2 | [vegetative to reproductive phase transition of meristem](https://www.ebi.ac.uk/QuickGO/term/GO:0010228) | Mediated mRNA m6A demethylation stabilizes the mRNA of the key flowering time regulators *FT* |
| *COR27* | *COLD REGULATED GENE 27* | DN90746_c0_g2 | regulation of circadian rhythm; response to cold; vegetative to reproductive phase transition of meristem | Bind to the chromatin of *TOC1* and *PRR5* to repress their transcription, regulate the circadian clock as well as freezing tolerance and flowering time |
| *YAB4* | *YABBY 4* | DN86923_c0_g2 | regulation of shoot apical meristem development | Over-expression in rice resulted in delayed flowering |
| *BAG6* | *BCL-2-ASSOCIATED ATHANOGENE 6* | DN105744_c0_g1 | vegetative to reproductive phase transition of meristem | Co-chaperon. *bag6* knock-out lines exhibited early flowering and a branched inflorescence phenotype |
| *EMB 1507* | *EMBRYO DEFECTIVE 1507* | DN107640_c1_g1 | [vegetative to reproductive phase transition of meristem](https://www.ebi.ac.uk/QuickGO/term/GO:0010228) | Affects flowering time via *FLC* splicing. |
| *FD.1* | *FLOWERING LOCUS D* | DN98102_c0_g1 | vegetative to reproductive phase transition of meristem; histone deacetylation | Probable histone demethylase that promotes flowering independently of the photoperiod and vernalization pathways by repressing *FLC* |
| *LDL2* | *LSD1-LIKE2* | DN107988_c2_g1 | [histone H3-K4 methylation](https://www.ebi.ac.uk/QuickGO/term/GO:0051568) | Probable histone demethylase that reduces the levels of histone H3 'Lys-4' methylation in chromatin of the floral repressor *FLC* |
| *CEN* | *CENTRORADIALIS* | DN37301_c0_g1 | negative regulation of flower development; vegetative to reproductive phase transition of meristem | May form complexes with phosphorylated ligands by interfering with kinases and their effectors (by similarity). Can substitute for *TFL1* |
| *ATH1* | *HOMEOBOX GENE ATH1* | DN108935_c1_g2 | floral organ abscission; photomorphogenesis; vegetative to reproductive phase transition of meristem | Transcription factor, which may be involved in the signal transduction pathway downstream of the *COP1*. Controls floral competency as a specific activator of *FLC* expression. Is responsive of the nuclear import of *STM* |
| *FT1* | *FLOWERING LOCUS T1* | DN104463_c2_g3 | photoperiodism, flowering | Regulation of flower development |
| *FT2* | *FLOWERING LOCUS T2* | DN87300_c2_g1 | flowering; photoperiodism; positive regulation of flower development; | Promotes the transition from vegetative growth to flowering. In onion acts as “florigen” |
| *DME* | *DEMETER* | DN107926_c1_g1 | DNA methylation; regulation of gene expression by genetic imprinting; vegetative to reproductive phase transition of meristem | Transcriptional activator involved in gene imprinting. Required for stable reproducible patterns of floral and vegetative development |
| *GTG2* | *GPCR-TYPE G PROTEIN 2* | DN96139_c0_g2 | vegetative to reproductive phase transition of meristem | Mutants showed early flowering phenotype |
| *FT3/5* | *FLOWERING LOCUS T 3/5* | DN93135_c0_g3 | regulation of flower development | In barley, *FT3* controls spikelet initiation but not floral development. |
| *TSF* | *TWIN SISTER OF FT* | DN105192_c3_g1 | photoperiodism, flowering; positive regulation of flower development | Acts as a floral pathways integrator redundantly with *FT*. *TSF* expression was induced rapidly upon activation of *CO* |
| *HMGB8* | *HIGH MOBILITY GROUP 8* | DN68376_c0_g1 | vegetative to reproductive phase transition of meristem | Plants with reduced amounts of *HMGB8* display various defects in vegetative and reproductive development, show early bolting, have abnormal flower. The early flowering of the mutant plants is associated with reduced expression of the floral repressor *FLC* |
| *EAT 3* | *TARGET OF EARLY ACTIVATION TAGGED 3* | DN95384_c0_g1 | [vegetative to reproductive phase transition of meristem](https://www.ebi.ac.uk/QuickGO/term/GO:0010228) | Probably acts as a transcriptional activator. Binds to the GCC-box pathogenesis-related promoter element. May regulate negatively the transition to flowering time and confers flowering time delay |
| *OTS2* | *OVERLY TOLERANT TO SALT 2* | DN109335_c2_g12 | [vegetative to reproductive phase transition of meristem](https://www.ebi.ac.uk/QuickGO/term/GO:0010228) | Protease that catalyzes two essential functions in the SUMO pathway. Regulates salt stress responses and flowering time |
| *AGL16* | *AGAMOUS-LIKE 16* | DN98786_c1_g3 | vegetative to reproductive phase transition of meristem | Participates in the repression of *FT* expression and floral transition, by interacting closely with the *FLC-SVP* pathways |
| *ARF2* | *AUXIN RESPONSE FACTOR 2 (ARF2)* | DN102038_c1_g4 | [vegetative to reproductive phase transition of meristem](https://www.ebi.ac.uk/QuickGO/term/GO:0010228) | Can act as transcriptional activator or repressor. Formation of heterodimers with Aux/IAA proteins may alter their ability to modulate early auxin response genes expression. Promotes flowering, stamen development, floral organ abscission and fruit dehiscence. |
| *PHP* | *PLANT HOMOLOGOUS TO PARAFIBROMIN* | DN95331_c2_g8 | flower development; histone H3-K4 methylation; positive regulation of flower development; vegetative to reproductive phase transition of meristem | Involved in regulation of flowering time. Required for the expression of the flowering repressors *FLC* and MADS-box genes of the *MAF* family. Required for histone H3 trimethylation on 'Lys-4' (H3K4me3) at the *FLC* locus. |
| *J3* | *DNAJ HOMOLOG 3* | DN48854_c0_g2 | photoperiodism, flowering; positive regulation of flower development | Mediates the integration of flowering signals through its interaction with *SVP* |
| *MBR1* | *MED25 BINDING RING-H2 PROTEIN 1* | DN108958_c1_g9 | flower development; proteasome-mediated ubiquitin-dependent protein catabolic process; vegetative to reproductive phase transition of meristem | 3 ubiquitin-protein ligase that functions as regulator of MED25 stability by targeting MED25 for degradation in a RING-H2-dependent way. Proteasome-dependent degradation of MED25 seems to activate its function as positive regulator of *FT* |
| *PRX17* | *PEROXIDASE 17* | DN104788_c0_g1 | vegetative to reproductive phase transition of meristem | *AGL15,* a transcription factor known to control bolting and abscission, binds to the *PRX17* promoter and regulates its expression level |
| *HDA6* | *HISTONE DEACETYLASE 6* | DN95733_c0_g7 | vegetative to reproductive phase transition of meristem | Mutant displayed a late-flowering phenotype. *flc* double mutants flowered earlier than *had6* plants, indicating that the late-flowering phenotype of *had6 is FLC-*dependent |
| *ASHH1* | *HISTONE LYSINE N-METHYLTRANSFERASE ASHH1* | DN92092_c0_g2 | flower development; histone methylation; regulation of transcription, DNA-templated;  response to UV-B; vegetative to reproductive phase transition of meristem | Involved in regulation of flowering time. Required for the expression of the *SOC1/AGL20*. Required for histone H3 trimethylation on 'Lys-4' (H3K4me3) at the *SOC1* locus |
